# Hollow Gradient-Structured Iron-Anchored Carbon Nanospheres for Enhanced Electromagnetic Wave Absorption

**DOI:** 10.1007/s40820-022-00963-w

**Published:** 2022-12-06

**Authors:** Cao Wu, Jing Wang, Xiaohang Zhang, Lixing Kang, Xun Cao, Yongyi Zhang, Yutao Niu, Yingying Yu, Huili Fu, Zongjie Shen, Kunjie Wu, Zhenzhong Yong, Jingyun Zou, Bin Wang, Zhou Chen, Zhengpeng Yang, Qingwen Li

**Affiliations:** 1 Key Laboratory of Multifunctional Nanomaterials and Smart Systems, Advanced Materials Division, Suzhou Institute of Nano-Tech and Nano-Bionics, Chinese Academy of Sciences, Suzhou, 215123 Jiangsu People’s Republic of China; 2 School of Science, Nanchang Institute of Technology, Nanchang, 330099 Jiangxi People’s Republic of China; 3 Division of Nanomaterials and Jiangxi Key Lab of Carbonene Materials, Jiangxi Institute of Nanotechnology, Nanchang, 330200 Jiangxi People’s Republic of China; 4 School of Nano-Tech and Nano-Bionics, University of Science and Technology of China, Hefei, 230026 Anhui People’s Republic of China; 5 School of Materials Science and Engineering, Nanyang Technological University, 50 Nanyang Avenue, Singapore, 639798 Singapore; 6 Jiangsu Key Laboratory of Micro and Nano Heat Fluid Flow Technology and Energy Application, School of Physical Science and Technology, Suzhou University of Science and Technology, Suzhou, 215009 People’s Republic of China; 7 School of Mechanical and Power Engineering, Nanjing Tech University, Nanjing, 211800 People’s Republic of China; 8 Henan Key Laboratory of Materials On Deep-Earth Engineering, School of Materials Science and Engineering, Henan Polytechnic University, Jiaozuo, 454003 People’s Republic of China; 9 College of Safety Science and Engineering, Xi’an University of Science and Technology, Xi’an, 710054 People’s Republic of China

**Keywords:** Gradient structures, Carbon nanospheres, Electromagnetic wave absorption, Impedance matching

## Abstract

**Highlights:**

- Microwave absorber with nanoscale gradient structure was proposed for enhancing the electromagnetic absorption performance.
- Outstanding reflection loss value (−62.7 dB), broadband wave absorption (6.4 dB with only 2.1 mm thickness) in combination with flexible adjustment abilities were acquired, which is superior to other relative graded distribution structures.
- This strategy initiates a new method for designing and controlling wave absorber with excellent impedance matching property in practical applications.

**Abstract:**

In the present paper, a microwave absorber with nanoscale gradient structure was proposed for enhancing the electromagnetic absorption performance. The inorganic–organic competitive coating strategy was employed, which can effectively adjust the thermodynamic and kinetic reactions of iron ions during the solvothermal process. As a result, Fe nanoparticles can be gradually decreased from the inner side to the surface across the hollow carbon shell. The results reveal that it offers an outstanding reflection loss value in combination with broadband wave absorption and flexible adjustment ability, which is superior to other relative graded distribution structures and satisfied with the requirements of lightweight equipment. In addition, this work elucidates the intrinsic microwave regulation mechanism of the multiscale hybrid electromagnetic wave absorber. The excellent impedance matching and moderate dielectric parameters are exhibited to be the dominative factors for the promotion of microwave absorption performance of the optimized materials. This strategy to prepare gradient-distributed microwave absorbing materials initiates a new way for designing and fabricating wave absorber with excellent impedance matching property in practical applications.
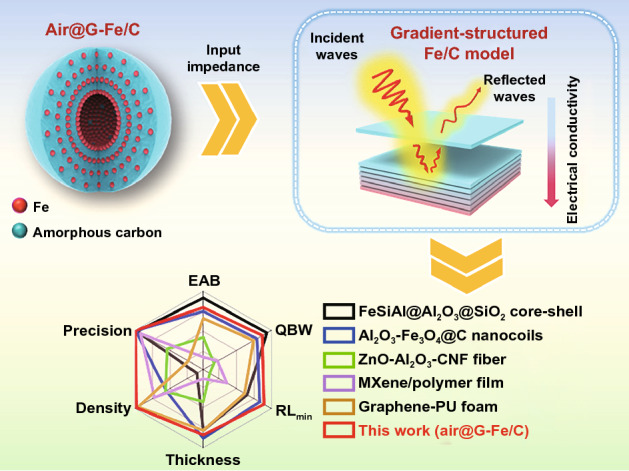

**Supplementary Information:**

The online version contains supplementary material available at 10.1007/s40820-022-00963-w.

## Introduction

The electromagnetic contamination caused by the extensive use of communication equipment has become one of the greatest endangers to human beings, creating a sharp requirement for electromagnetic-absorbing (EMA) material with excellent performances [1]. Carbon-based materials (CBMs), such as carbon fibers [2], carbon nanotubes [3], carbon nanosphere [4] as well as graphene [5], are considered ideal candidates for EMA because of their excellent traits of lightweight, corrosion resistance and excellent dielectric attenuation. Nevertheless, the EMA properties of single CBM absorbers are severely affected by unsatisfied impedance and inferior broad-frequency absorption ability [6, 7]. Generally, two typical strategies, such as integrating carbon with magnetic materials and structure design [8], are applied to settle these dilemmas. For improved impedance matching and highly adjustable polarization, exploiting multicomponent tactics has proven to be one of the most effective ways [9]. However, simply assembling magnetic components (MCs) with CBM cause the aggregation phenomenon, resulting in magnetic attenuation and oxidative deterioration [10]. Thus, many recent works were conducted to find out the relationship between microstructural design and EMA performance, due to structure-induced physical effects [11, 12]. For example, structures of single atoms [13], heterointerface [14], phase engineering [15], hollow spheres [16], cellular structure [17] and nanofiber [18] are proved to be helpful in improving EMA ability. However, exploiting an effective method to integrate all the merits of prominent impedance matching, lightweight, chemical resistance, and anti-agglomeration in a single structure is still challenging.

Recent years have been seen enormous efforts to designing a structure in multi-scales to acquire the ideal physical properties simultaneously [19–22]. Especially, combining gradient structures with others is considered to be an ideal method to solve the problems of multi-target, for the excellent “absorption–reflection–reabsorption” effect and eminent intensifying of incidence microwave [15, 23]. Moreover, the response frequency and intensity can be regulated by simply tuning the functional layer's constitutes and thickness [24]. Plenty of explorations certify the gradient structure with gradually increasing electro-conductibility is an effective way to adjust the EMA performance [25–27]. However, till now, the introduction of gradient components is stepwise, complicated, and time/energy**-**consuming. Moreover, the precisely fabricating gradient structures (i.e., multilayered and gradient films) in a simple way at the atomic scale have rarely been reported, and the consequence of dispersion, ingredient, interface, and imperfection on the microwave absorption has not been clarified yet. Hence, developing new regulating methods to prepare multilayered components with gradient distribution is meaningful. Fortunately, the inorganic–organic competitive coating (I-OCC) strategy shows the unique superiorities in building gradient components [28]. Because of the adjustable microstructure, convenient components as well as precise design dimensions, I-OCC methods can be used to synthesize EMA material with both magnetic-dielectric synergistic effect and excellent impedance matching performance. Furthermore, dielectric attenuation property could be elevated via carbonizing the framework precursors by designing different alloy or metal components, such as Fe, Cu, Ag, Co and Ni. However, researches on EMA absorbers with combined features of atom-level gradient and hollow structure are seldom explored.

Herein, an as-made Fe gradient-structured nanospheres (SiO_2_@G-Fe/C) were resoundingly fabricated for a magnetic–dielectric synergy EMA materials via the I-OCC method. Under solvothermal treatment, abundant Fe_3_O_4_ nanoparticles (~ 10 nm) were gradient-dispersed in the polymer carbon shell, forming gradually increasing electrical conductance and heterogeneous interfaces in dielectric shell precursor. For maximum polarization, impedance matching and light weight, the complex permittivity of SiO_2_@G-Fe/C nanospheres could be modified by adjusting the SiO_2_@G-Fe_3_O_4_/C precursor via annealing polymer carbon/Fe_3_O_4_ and etching SiO_2_ core (Table S1). The microwave absorber was acquired by hollow gradient-structured iron-anchored amorphous carbon nanospheres (air@G-Fe/C). Based on the multiple reflection effects, air@G-Fe/C nanospheres show outstanding EMA properties in the reflection damping performance, broadband absorbing ability with peak RL intensity of -62.7 dB at merely 2.1 mm and the effective absorbing bandwidth (EAB) indeed up to 6.4 GHz. The hollow gradient-structured iron-anchored amorphous carbon nanospheres establish fascinating microwave absorbing construction with the following merits: above all, the gradient-structured Fe NPs dispersed in the carbon shell (~ 100 nm) bring about both low-to-high charge density distribution from out to inside and magnetic coupling among micro-balls, which was verified by the electron holography analysis, supplying superior impedance matching, chemical stability, anti-agglomeration and lightweight for the EMA behaviors. Meanwhile, more substantial polarization was formed after orderly anchoring abundant Fe NPs in amorphous hollow carbon with different electro-negativity improved magnetic damping. Moreover, magnetic–dielectric synergistic effect and multiple-reflection of hollow air@G-Fe/C nanospheres will cause an ultra-wide frequency microwave responding and lightweight performance. The gradient distribution of electrical conducting structure at the atomic scale might offer a novel way to elucidate the relationship between macroscopic dielectric characteristics and the atomic-scale constructions for future fabrication of EMA material.

## Experimental and Calculation

### Materials

SiO_2_ nanoballs (~ 300 nm) were purchased from Beijing Zhongkeleiming Daojin Technology Co. Ltd. Ferrocene (FeC_10_H_10_, 98%) and acetone (CH_3_COCH_3_, 99.9%) were obtained from Sinopharm Chemical Reagent Co., Ltd. Hydrogen peroxide solution (H_2_O_2_, 25%) were acquired from Shanghai Macklin Biochemical Co., Ltd. Sodium hydroxide pellets (NaOH, 98%) were obtained from Aladdin Reagent (Shanghai) Co., Ltd.

### Synthesis of Air@G-Fe/C Composite

The hollow gradient-structured iron-anchored amorphous carbon nanoballs were synthesized via the inorganic–organic competitive coating method. Homogeneous SiO_2_ nanoballs with the size of ~ 308 nm were offered as the template core (Fig. S1). Firstly, mixtures of 0.20 g SiO_2_ nanoballs, 0.40 g ferrocene and 25 mL of acetone were stirred for 10 ~ 20 min. Then, 2.0 mL of H_2_O_2_ solution was added to the compound. After sustainability blending for 30 ~ 50 min, the hybrids were decanted into a hydrothermal reactor and heated at 210 °C for 1 day. The outcome was assembled by centrifugation and washed by deionized water and ethanol four times, respectively. Then, this precursor was dried overnight and followed heated at 700 °C in argon for 30–60 min. At last, the gradient-structured Fe/C nanoballs were gained via getting rid of the SiO_2_ cores with NaOH aqueous solution; the product obtained was denoted as air@G-Fe/C. For comparison, composites with different Fe dispersions were prepared and their synthesis processes were the same as air@G-Fe/C, except for solvothermal process. The final composites were marked as T180, T190, T200 and T210, corresponding to the solvothermal temperature of 180, 190, 200 and 210 °C, respectively. In order to research the carbonization effects on dielectric loss ability of composites, samples with the same prepared procedures but different carbonization temperatures were also synthesized. The final composites were denoted as T600, T700, T800 and T900, corresponding to the carbonization process of 600, 700, 800, and 900 °C, respectively.

### Characterization

The cross-sectional scanning electron microscopy (SEM) samples were managed with the focused ion beam (FIB, FEI Helios NanoLab 600i). The distribution of iron in carbon shell was measured by auger electron spectrometer (AES, PHI 710) with coaxial tube mirror energy analyzer and electron backscatter diffraction detector. The surface morphology of SiO_2_ nanospheres, as-made Fe_3_O_4_ gradient-structured nanoballs and air@G-Fe/C nanosphere were analyzed via a scanning electron microscope (S-4800). Field-emission transmission electron microscope (FETEM) and high-resolution TEM (HRTEM) images were characterized using JEOL JEM-2100F. Energy-dispersive X-ray spectroscopy (EDS) and elemental mapping were performed using an Ultim Max TLE laser-cooled silicon drift detector (SDD, OXFORD Instruments). Electron holographic information was operated by a FETEM (JEOL, JEM-2100F) with a postcolumn Gatan imaging filter (GIF, Tridium 863) system working at 200 kV. Powder X-ray diffraction (PXRD) spectrum was obtained using an X-ray diffractometer (Bruker D8 Advance) using CuKα as the irradiation source (λ = 1.54060 Å). Raman patterns of powder samples were obtained on Horiba (LabRAM HR Evolution) with a laser excitation wavelength of 514.5 nm. Fourier transform infrared (FT-IR) spectroscopy was characterized with a Nicolet In10. X-ray photoelectron spectroscopy (XPS) was conducted using scanning X-ray microprobe (Thermo Scientific K-Alpha +). The magnetic hysteresis loops were obtained via LakeShore vibrating sample magnetometer (VSM). Nitrogen adsorption–desorption isotherm was characterized by an accelerated surface area and pore size analyzer (ASAP, Micromeritics TriStar II 3020). The pore size distribution was analyzed via Barrett–Joyner–Halenda (BJH) strategy, and data were analyzed by software BELMaster (Ver 7.1.1.0, MicrotracBEL Corp). Thermogravimetric analysis (TGA) was measured by a thermal gravimetric analyzer (Labsysevo) in the air from 50 to 900 °C with a heating rate of 10 °C/min. Electromagnetic parameters were characterized by a PNA Microwave network analyzer (Keysight, N5227A) in the scope of 2–18 GHz. The measured materials were prepared by homogeneously mixing the absorbents with paraffin matrix by the mass fraction of 40% and compacted into a coaxial ring of 3.5 mm outer diameter and 1.52 mm inner diameter. Autolab PGSTAT302N electrochemical workstation was employed on electrochemical impedance spectra (EIS) for analyzing charge carrier behavior.

## Results and Discussion

### Preparation of Air@G-Fe/C Nanosphere

**Fig. 1:**
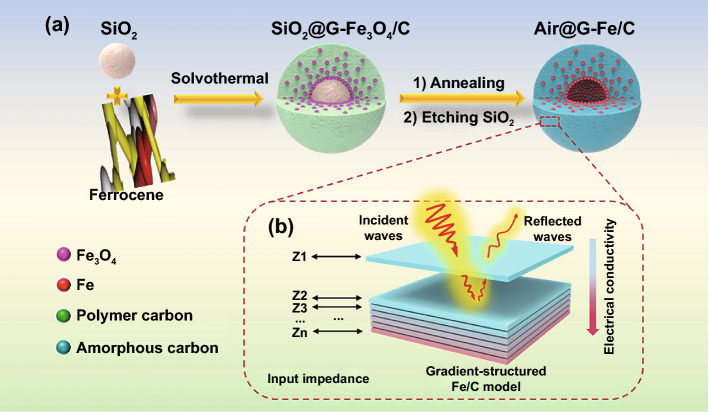
**a** Schematic diagram of the preparation course and **b** electromagnetic wave absorption mechanism of air@G-Fe/C

**Fig. 2:**
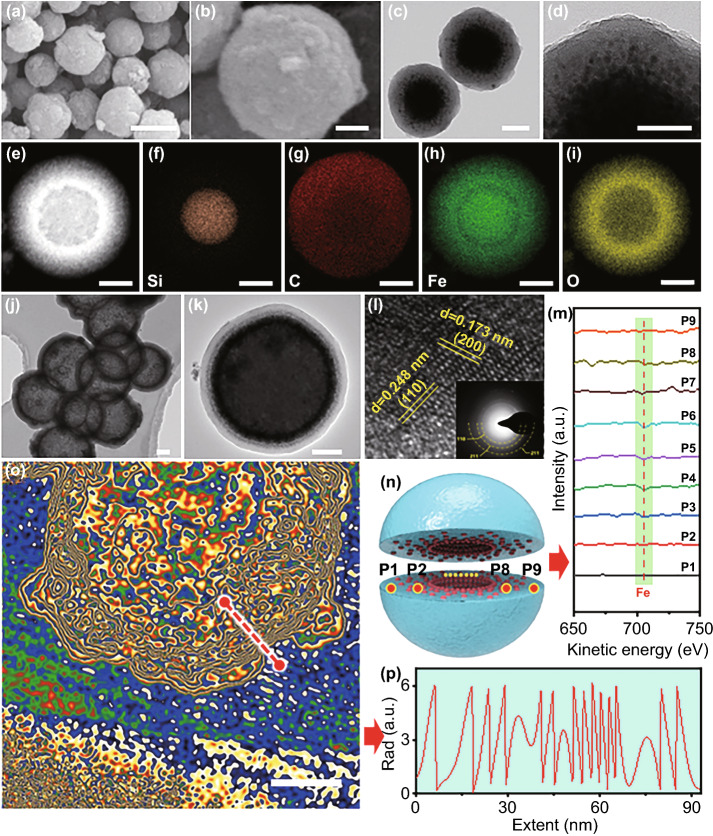
Morphologies and dispersion measurement of the grade-distributed Fe_3_O_4_/C nanospheres precursor and air@G-Fe/C nanospheres of T210. **a, b** SEM and **c, d** TEM images of the graded distributed Fe_3_O_4_@C nanospheres precursor. **e** STEM and **f-i** the corresponding EDS mapping images of Si, C, Fe, and O elements dispersion in the typical nanospheres. **j, k** TEM images of the hollow graded distributed Fe_/_C nanospheres. **l** HRTEM images of graded distributed Fe_/_C nanospheres, the inset in (**l**) is SAED image of the hollow graded distributed Fe_/_C nanospheres. **m** Auger electron spectroscopy (AES) of Fe element and **n** the corresponding section schematic. **o** The off-axis electron holography and **p** corresponding charge density distribution line profiles obtained from the red line in (**o**). The scale bar of **a** is 500 nm, the scale bar of **b, c**, and **d** is 100 nm, the scale bar of **e-i** is 200 nm, the scale bar of **j, k**, and **o** is 100 nm

The preparation procedure of the air@G-Fe/C nanosphere is presented via I-OCC and deposition process, as shown in Fig. 1. In this process, ferrocene is employed as the single donator for both carbon and Fe to wrap Fe_3_O_4_/C coat on the surface of SiO_2_ nanosphere with ~ 300 nm (Fig. S1). Field-emission scanning electron microscopy (FESEM) pictures reveal that solvothermal temperature has a faint influence on the diameter of the as-made core–shell nanospheres, all of which are uniform with a size of ~ 500 nm (Fig. 2a-b and S2). Compared with initial SiO_2_ cores, the thickness of the gradient-structured nanospheres increases, implying that the shell thickness is about 100 nm (Fig. S3a-d). Different from the thickness of Fe_3_O_4_/C coating, the distribution of Fe_3_O_4_ NPs anchored in polymer carbon shell is dependent on the inorganic–organic competitive coating and deposition process, which is determined by thermodynamics of the solvothermal course. In this process, the iron ions and carbonaceous specie, which are hydrolyzed from ferrocene, have a competitive deposition reaction when hydrolyzed into iron oxides and polymerized into an amorphous carbon layer (Fig. S4). At a relatively low temperature (i.e., 180 °C), iron ions could not fully be dissociated from coordination of both cyclopentadienes and hydrolyzed, thus hindering the formation of iron oxide species. However, cyclopentadiene disintegrated from ferrocene could be polymerized into amorphous carbonaceous and covered the surface of silica nanospheres actuated via the affinity interaction. Hence, the uniform SiO_2_@polymer carbon core–shell structure is acquired (Fig. S3a, e). When temperature is higher (190 ℃), the polymerization reaction of carbonaceous species is slightly accelerated, forming thick carbon layer on the surface of SiO_2_ at first (Fig. S4b). In this condition, the nucleation and growth of Fe_3_O_4_ NPs could be activated by the cross-bonding, and hydrolyzation of Fe ions was disintegrated from ferrocene. Simultaneously, an ultrathin carbon layer is formed on the surface of SiO_2_@C nanospheres, generating an island-like Fe_3_O_4_ NPs morphology on the surface of ultrathin carbon cover (Figs. S3b, f, and S4b) [28]. Moreover, with the increase in solvothermal temperature (200 and 210 ℃), the nucleation and growth rate of Fe_3_O_4_ accelerate to quicker than that of polymerization, leading to a gradient anchor of Fe_3_O_4_ in the polymer carbon shell (Fig. S3c, d, g, and h). This phenomenon is verified by XRD patterns and FT-IR profiles as shown in Fig. S5. Then, a low-to-high gradient distribution of Fe_3_O_4_ NPs anchored in a polymer carbon shell can be obtained by a simple solvothermal reaction regulation. After the annealing process and selective etching of the SiO_2_ nano-ball core, the obtained hollow Fe/C nanospheres (air@G-Fe/C) with an inner void of ~ 300 nm still demonstrate mono-distributed diameter dispersion of ~ 500 nm with faintly crumpling (Fig. S3i-l). To the best of our knowledge, it is the first time to prepare hollow sphere EMA materials with increasing electrical conductance at the atomic level in a simple way.

### Characterization of Air@G-Fe/C Nanosphere

The morphology of gradient-structured Fe_3_O_4_/C nanospheres precursor (SiO_2_@G-Fe_3_O_4_/C) fabricated at 210 ℃ is revealed in Fig. 2a-b. The smooth surface with mild corrugation is derived from the intercalation and polymerization of cyclopentadiene species decomposed from ferrocene. The TEM images of SiO_2_@G-Fe_3_O_4_/C nanospheres render the distinct gradient structures (Fig. 2c-d). The Fe_3_O_4_ NPs (~ 10 nm) in carbonaceous shells are assembled into the sublayer with high-to-low constituent dispersion from inside to outside, meanwhile coated with an outer polymer coat (~ 30 nm) void of Fe_3_O_4_ NPs (Fig. 2d). High-resolution TEM (HRTEM) images and selected area electron diffraction (SAED) characterization display that the Fe_3_O_4_ NPs inside the polymer coat is altitudinally crystallized with a diameter of ~ 10 nm and completely coated via polymer carbon layers (Fig. S6). The scanning TEM (STEM) and corresponding EDS mapping images (Fig. 2e-i) present that the iron and oxygen elements in SiO_2_@G-Fe_3_O_4_/C nanospheres are gradient-dispersed in the outer polymer carbon shell, coinciding with the information displayed in Fig. 2c-d. SEM images and corresponding EDS mapping images of SiO_2_@G-Fe_3_O_4_/C precursor prepared by FIB also demonstrate the uniform coating of carbon layer on the surface of SiO_2_ (Fig. S7). The gradient distribution of iron components in T210 after the calcination and etching process can be verified via the TEM images at first (Figs. 2j-k and S8). HRTEM images and SAED characterizations reveal that the Fe_3_O_4_ is reduced to Fe. The iron component in the inner mesoporous carbon shell is also highly crystallized with a diameter of about 20 nm, conformably wrapped via graphitic carbon layers (Figs. 2l and S8–S9). Auger electron spectroscopy (AES) was used to characterize the gradient structure of Fe element in the air@G-Fe/C nanospheres (Fig. 2m-n). The HAADF and elemental mapping images of T210 also distinctly imply that the Fe component is gradient-implanted in the inner layer of the amorphous carbon coat (Fig. S10). Moreover, the successful fabrication of gradient-distributed Fe NPs can also be confirmed via the corresponding EDS line scan obtained from the HAADF image (Fig. S11). Off-axis electron holography (EH) characterization was performed for synthetical analysis of the relationship between gradient-structured Fe NPs [29], the dielectric polarization performance of air@G-Fe/C MA absorber, and off-axis electron holography (EH) characterization. Figure 2o reveals an electron hologram obtained by air@G-Fe/C nanospheres (T210) under a positive bias voltage of 200 kV; the electrostatic potential displays a situation of multiple layers of concentric rings. The possible electrostatic dispersion from Fig. 2o (as marked in the dotted red line) is reconstructed, revealing considerable polarization from inside to surface, as displayed in Fig. 2p. Therefore, all these information demonstrate that a hollow carbon shell with gradient-embedded ultrafine Fe particles can be precisely synthesized at ~ 20 nm. Compared to the traditional homogeneous dispersion structure, this structure might be favorable for both boosting ample heterogeneous interfaces for the improved interfacial polarization and reinforced impedance of absorbers (Fig. 1b).

**Fig. 3:**
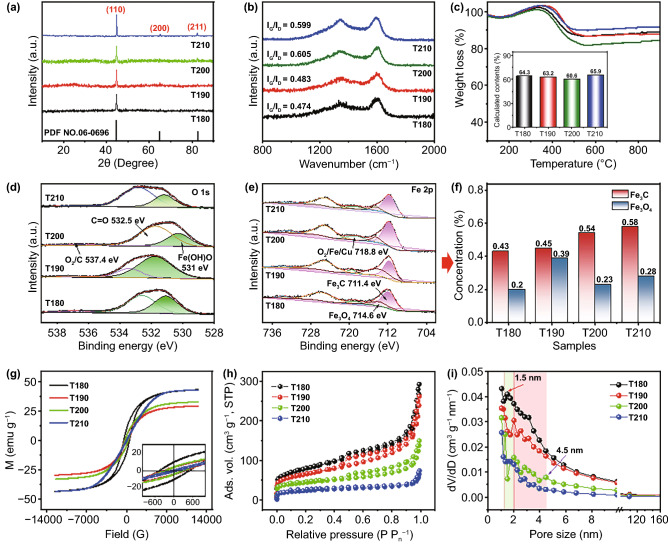
Compositional characterization of air@G-Fe/C nanospheres. **a** XRD patterns. **b** Raman spectrum. **c** TGA curves, Inset is the calculated contents of Fe. High-resolution XPS signals of **d** O 1*s* and **e** Fe 2*p*, and **f** the corresponding calculated concentration of Fe_3_C and Fe_3_O_4_. **g** Magnetic hysteresis loops at 298 K, Inset is the enlarged scale at the low field. **h** N_2_ adsorption–desorption isotherm and **i** pore size distribution

Intrinsically, EMA capability is closely correlated with the constituent of the absorber [30]. Thus, the composition of hollow air@G-Fe/C nanospheres prepared by different solvothermal temperatures (180, 190, 200, and 210 ℃) was investigated. Figure 3a presents the XRD spectrum of hollow air@G-Fe/C nanospheres. All samples display the same primary diffraction peaks positioned at 2θ = 44.7°, 65°, and 82.3°, corresponding to (110), (200), and (211) lattice planes of body-centered cubic iron phase based on JCPDS No. 06–0696. Figure 3a reveals that solvothermal temperature has little influence on the formation of Fe NPs. Raman spectra show the D and G bands of amorphous carbon at 1351 and 1585 cm^−1^ in air@G-Fe/C nanospheres (Fig. 3b). The *I*_*G*_*/I*_*D*_ peak intensity ratios of T180, T190, T200, and T210 are 0.474, 0.483, 0.605, and 0.599, respectively. A higher ratio reveals a mildly more graphitic carbon and mispairing within the hollow amorphous carbon shell; this performance can be ascribed to the Fe-induced graphitization mechanism in the course of pyrolysis [31] and is useful for the incidence and attenuation of the electromagnetic wave in the absorber. Moreover, there are no significant Fe characteristic bands in the frequency of 800–1000 cm^−1^; this also reveals that Fe NPs have been totally coated [32]. The iron concentration of air@G-Fe/C nanosphere was assessed by TGA measurements (Fig. 3c). As ferroferric oxide is the single residue after the combustion of air@G-Fe/C nanospheres; Fe contents can be figured out via Eq. (1) [33]:

1 $$ {\text{wt}}\% \, \left( {{\text{Mo}}} \right) = {\text{wt}}\% \, \left( {{\text{residual}}\;{\text{mass}}} \right) \times 3M\left( {{\text{Fe}}} \right)/M\left( {{\text{Fe}}_{3} {\text{O}}_{4} } \right) $$

where M(Fe) and M(Fe_3_O_4_) are the molecular weight of iron and ferroferric oxide. The corresponding Fe concentrations in T180, T190, T200 and T210 were computed to be 64.3, 63.2, 60.6 and 65.9 wt%, exhibiting a similar Fe anchoring ability.

**Fig. 4:**
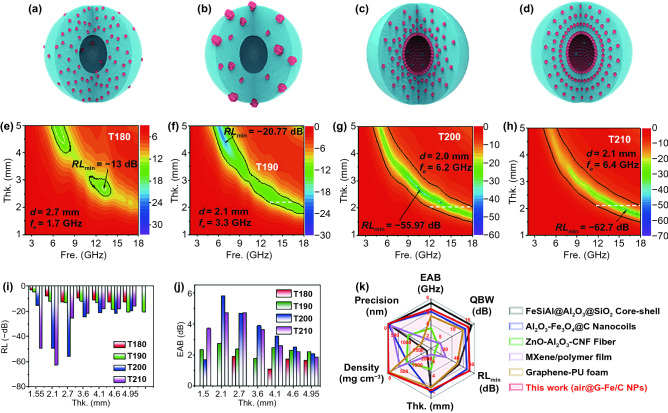
The schematic diagram and RL values of **a, e** T180, **b, f** T190, **c, g** T200, and **d, h** T210. Comparison of the **i** RL and **j** EAB of different samples. **k** Radar chart for comparison of EAB, QBW, RL_min_, thickness (abbreviated as Thr.), density, and fabrication precision in the optimized T210 and the other reported gradient distributions/layers structures. Frequency is abbreviated as Fre

The elemental compositions and chemical state of air@G-Fe/C nanospheres were further analyzed by X-ray photoelectron spectroscopy. The wide-scan survey spectrum via XPS presents the existence of Fe, O and C elements (Fig. S12a). The high-resolution C 1*s* pattern is displayed in Fig. S12b, and the main bonds at 284.8 eV uncover the graphite carbon that is the dominant component of samples as well [34]. The O 1*s* spectra are generally used to elucidate the disparate chemical states of O (Fig. 3d). The spectra can be deconvolved into three sub-constituents, and the two dominating peaks at 531 and 532.5 eV are ascribed to Fe(OH)O and C = O bonds, respectively. Compared with T180 or T190, T200 and T210 display a feebler intensity of Fe(OH)O bond, attributing to the preferential coating of Fe NPs. Figure 3e shows that the high-resolution XPS of Fe 2*p* can be fitted into four components, and the three primary peaks at 711.4, 714.6, and 718.8 eV correspond to the Fe_3_C, Fe_3_O_4_, and O_2_/Fe/Cu, respectively. Due to the higher induced graphitization phenomenon caused by uniformly dispersed Fe [33], the composition of Fe_3_C in T200 and T210 increased to 54% and 58%, which is consistent well with the results displayed in Fig. 3b. T200 and T210 demonstrate lower Fe_3_O_4_ contents than T190, confirming the evidently impeding the oxidation in the gradient structure. To understand the existing state, species, and relative ratios of O 1*s* and Fe 2*p*, the high-resolution curve of air@G-Fe/C samples was ulteriorly calculated and is exhibited in Fig. 4f and Table S2.

**Fig. 5:**
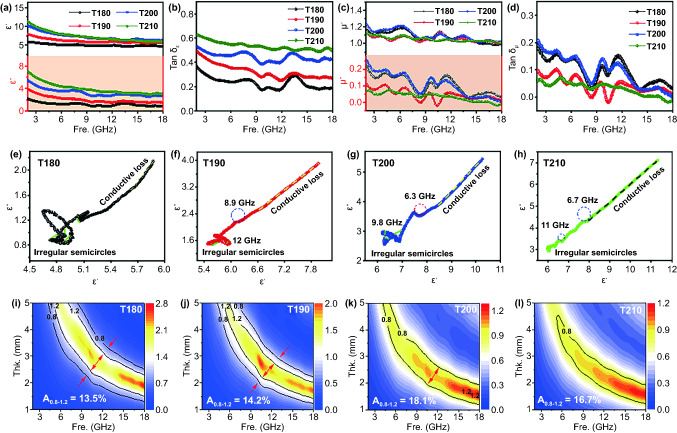
Electromagnetic parameters and calculated |*Z*_in_/*Z*_0_| value maps of different samples. **a** ε′ and ε′′. **b** The tan*δε* plots (tan*δε* = *ε''/ε'*). **c** µ′ and µ′′. **d** Magnetic loss tangent (tan*δε* = *μ''/μ'*). Cole–Cole plots of **e** T180, **f** T190, **g** T200, and **h** T210. Calculated impedance matching degree |*Z*_in_/*Z*_0_| maps of **i** T180, **j** T190, **k** T200, and **l** T210

The EMA performance is also strongly linked with the magnetic characteristic and porous structures [35, 36]. Basically, a higher magnetization capacity leads to a more substantial magnetic attenuation property. Figure 3g presents the magnetic hysteresis loops of the air@G-Fe/C nanosphere. Notably, the nanospheres display a typical ferromagnetic hysteresis characteristic with saturation magnetization (*M*_s_) being 43.4, 29.5, 32.7, and 43.0 emu g^−1^, and the coercive fields (*H*_c_) of 481.2, 348.6, 279.1, and 104.4 G (Fig. 5g), respectively. The remanence magnetizations (*M*_r_) of T200 and T210 reduce to 4.16 and 1.15 emu g^−1^, which are much lower than that of bulk iron (*M*_s_ ~ 200 emu g^−1^) due to the existence of nonmagnetic carbon in nanospheres. Compared with bulk materials, NPs usually demonstrate superparamagnetic capabilities caused by magnetocrystalline anisotropy [37]. In this work, the magnetic Fe NPs in T190 were aggregated on the surface of the nanosphere, resulting in a smaller *M*_s_ value. Besides, Fe NPs in T190 were easier to be oxidized, resulting in the intention of saturation magnetization abated, which might be another reason for the minimizing of *M*_s_. T200 displays a downtrend of *M*_s_, which might be resulted from a fewer iron content (inset in Fig. 3c). The unique pore structures in the hollow air@G-Fe/C nanospheres offer ample surface and interfaces among air, iron and carbon, as revealed by the BET characterization. The specific surface area of samples up to 281.74, 229, 156.52, and 83.054 m^2^ g^−1^ would benefit electromagnetic wave dissipation by boosting the impedance matching and offering different interfacial polarization. It is demonstrated that whole air@G-Fe/C nanospheres present both mesoporous and microporous characteristics (Fig. 3h-i). The mean pore diameters of air@G-Fe/C nanospheres fall in the range of 5.05–6.91 nm, demonstrating the dominant mesoporous (Table S3). Therefore, the iron-amorphous carbon structure can be finely fabricated to hamper the oxidation and agglomeration of iron when making use of different existing structures of carbon and iron, which is convenient to adjust the dielectric characteristic and easy to scale up, opening a novel way to limit sacrifices of magnetic loss and improve the incidence of the microwave.

### Microwave-Absorbing Ability of Air@G-Fe/C Nanospheres

The reflection loss (RL) values are significant for evaluating the EMA performance [38]. The structure illustrations of different samples are shown in Fig. 4a-d. Figure 4e-h reveals the RL maps of air@G-Fe/C nanospheres in different frequencies (2.0–18.0 GHz) and thicknesses (1.0–5.0 mm). Since the gradually increasing electro-conductibility construction resulted from gradient-distributed Fe nanoparticles, T200 and T210 show better RL properties than T180 and T190 (Fig. S13). The peak reflection loss performances that T180 and T190 offer just reaches -13 dB at 13.4 GHz with 2.7 mm thickness (Fig. 4e) and − 20.77 dB at 6.1 GHz with 2.1 mm thickness (Fig. 4f), respectively. Nevertheless, T200 shows the peak reflection loss intensity of -55.97 dB at 10.5 GHz with just 2.7 mm thickness, and the EAB ability contains 6.2 GHz (range from 11.8 to 18 GHz) with just 2.0 mm thickness (Fig. 4g). T210 demonstrates the most vigorous absorption intensity of − 62.7 dB at 15.8 GHz with 1.95 mm thickness and the adequate absorbing bandwidth from 11.6 to 18 GHz (i.e., 6.4 GHz) at merely 2.1 mm thickness (Fig. 4h). Compared with T180, the strongest RL of T200 and T210 are increased by 4.3 and 4.82 times, respectively. The RL histogram distribution maps could obviously reveal the electromagnetic wave absorption capacity of different materials. The outcome demonstrates that T200 and T210 have a more excellent EMA abilities than T180 and T190 (Fig. 4i) in the range of 1.5–4.95 mm. Figure S14a-d presents the electromagnetic wave absorption capacity of air@G-Fe/C nanospheres in the range of 1–5 mm. It is shown that the respective RL intensity greater than -10 dB for T200 and T210 covers the range as broad as 4–18 GHz and 4.15–18 GHz, covering the whole C band, X band and Ku band. This phenomenon demonstrates that a perfect RL intensity and broadband absorbing characteristic could be acquired by regulating the distributed structure of Fe nanoparticles in air@G-Fe/C nanospheres.

EAB and integrated qualified bandwidth (QBW) are vital parameters to assess the wideband property of electromagnetic wave absorbers. Generally, the receivable reflection loss intensity for EAB and QBW is -10.0 dB, representing that the input electromagnetic wave is damped by 90% [39]. The integrated QBW values represent the ability of absorber in the range of 1–5 mm. As shown in Fig. 4j, T200 and T210 demonstrate an excellent wideband characteristic than T180 and T190, almost in all calculated thickness ranges (1.5–4.95 mm). More specifically, if the thickness is 2.1 mm, the EAB for T200 and T210 is 5.8 GHz and 4.7 GHz, broader than T180 (0 GHz) and T190 (2.1 GHz). The integrated qualified bandwidth of T180, T190, T200, and T210 achieves 4.32, 12.9, 14, and 13.85 GHz, separately, corresponding to the thickness from 1.0 to 5.0 mm (Fig. S14e). Furthermore, the EAB, integrated QBW, *RL*_min_, thickness, density, and fabrication precision of T210, along with some conventional gradient distribution/multilayer structures, are exhibited in Fig. 4k. In comparison, air@G-Fe/C nanospheres reveal an outstanding broadband EMA performance with the integrated qualified bandwidth covering all of the Ku-band, X-band, C-band, and even part of the S-band. Apart from wide integrated qualified bandwidth, strong reflection loss performance with smaller thickness, lower density, and finer synthesis accuracy could also be satisfied via air@G-Fe/C absorber, indicating its excellent comprehensive performance in the application of microwave absorption territory. More detailed comparative information on air@G-Fe/C absorber and other conventional gradient distribution/multilayer composites are listed in Table S4 [40–46].

### Modulation Mechanism of Air@G-Fe/C Nanospheres

The EMA ability is synergistically determined by dielectric attenuation, magnetic attenuation, and impedance matching traits. To reveal the intrinsic electromagnetic wave absorbing mechanism of air@G-Fe/C nanospheres, the primary electromagnetic parameters (*ε*_*r*_, *μ*_*r*_) are analyzed first. Figure 5a shows the *ε′* and *ε″* of relative complex permittivity for air@G-Fe/C nanospheres in the frequency from 2 to 18 GHz. T180 and T190 are exhibited to be a weaker *ε′* and *ε″* than T200 and T210. However, according to Weston’s theorem, stronger permittivity generates senior damping ability, which might cause an inferior impedance matching feature. Thus, the tan*δ*_*ε*_ of relative complex permittivity is further investigated. As shown in Fig. 5b, the tan*δ*_*ε*_ is in the sequence of T210 > T200 > T190 > T180 from 2.0 to 18.0 GHz, which is in line with the results of *RL* values. Typically, magnetic dissipation also plays a crucial character in the EMA procedure, which could be adjusted via its complex permeability and intrinsic magnetic capacity. It is revealed that whole air@G-Fe/C nanospheres present a similar *µ′* and *µ″* values of approximately 1.2 and 0.15, respectively, for the distribution difference of Fe nanoparticles (Fig. 5c). The corresponding magnetic loss tangent factors represented in Fig. 5d are smaller than tanδ_ε_, uncovering the slender magnetic loss ability in the EMA process. Meanwhile, the absolute value of tan*δ*_*ε*_/tan*δ*_*μ*_ curve is analyzed to further estimate the attenuation character between dielectric and magnetic factors (Fig. S15). Obviously, the importance of tan*δ*_*ε*_/tan*δ*_*μ*_ for T210 and T200 is higher than that of T180 or T190 in the scope of 12–18 GHz, proposing the dominative role of dielectric attenuation ability in high frequency. It is interesting that value of tan*δ*_*ε*_/tan*δ*_*μ*_ for T210 reaches about 10 in the range of 2–7 GHz, which ought to be determined by magnetic loss other than dielectric loss, demonstrating a possibility for improving EMA ability in low frequency by regulating dielectric parameter, consistent with our previous work [47]. Moreover, the eddy current coefficient (*C*_*0*_) diminishes and then keeps stabilization from 7.5 GHz, demonstrating the natural syntony of the magnetic zero-valent Fe NPs, and the exchange resonance could be ignored (Fig. S16a).

The attenuation constant (*α*) is evaluated by the transmission line theory. As displayed in Fig. S15B, all air@G-Fe/C nanospheres demonstrate a gradually increasing value in the range of 2.0–18.0 GHz. The intention of α is in the sequence of T210 > T200 > T190 > T180, which is absolutely consistent with the conclusion of *ε′* and *ε″* (Fig. 5a), yet verifying the dominative role of dielectric attenuation in the MA process of air@G-Fe/C nanospheres. To better understand dielectric attrition, the alternative conductivity (*δ*_*ac*_) has been analyzed based on *δ*_*ac*_ = *ε*_*0*_*ε″ω* = *2πfε*_*0*_*ε″*. As presented in Fig. S16c-d, the *σ*_ac_ parameters for T200 and T210 are greater than T180 or T190 in the frequency of 2.0–18.0 GHz, and their corresponding mean conductivities are 1.04, 0.99, 1.66, and 1.97 S cm^−1^, respectively, consistent with the results of electrochemical impedance spectra (Fig. S25a-b). Based on the free-electron mechanism (*ϵ″≈σ/2πϵ*_*0*_*f*), the electrical conductivity (*σ*) correlates positively with *ϵ″* values, which is consistent with the result shown in Fig. 5b. This characteristic stemmed from the fact that more interface polarization is indeed induced in T200 and T210 due to more defects between amorphous carbon and graphitic carbon abducted by the iron oxide (Fig. 3b). According to Debye's theory, dielectric loss performance primarily originated from polarization attenuation and conductivity attenuation. The conduction loss is ascribed to the migration of electrons in the 3D interconnected network structure and the carbon shells of the nanocapsules. The multiple polarization relaxations in the air@G-Fe/C include the dipolar polarizations and multiple heterogeneous interfacial polarizations [48]. Debye relaxation (Cole–Cole curves) can be denoted by Eq. (2) [49]:

2 $$ \left( {\varepsilon^{\prime } - \frac{{\varepsilon_{s - } \varepsilon_{\infty } }}{2}} \right)^{2} + \left( {\varepsilon^{\prime \prime } } \right)^{2} = \left( {\frac{{\varepsilon_{s - } \varepsilon_{\infty } }}{2}} \right)^{2} $$

In the Cole–Cole curve, every semicircle delegates a relaxation behavior. The dipole and interface polarity were primarily derived from asymmetric charges apportion at the defect, functional group, and the border of carbon and Fe. Figure 5e-h reveals the Cole–Cole plots of air@G-Fe/C nanospheres. T210 presents fewer irregular semicircles than T180 and T190. Nevertheless, T200 shows a distinct reverse trait with more semicircles than T190. As a primary method for attenuating the electromagnetic waves, the completely inverse behaviors with polarized characteristics illuminate that conductivity is the dominative element for the microwave loss ability of air@G-Fe/C nanospheres.

Impedance matching value (*Z*) is an essential ingredient for EMA ability, which indicates the property of microwave loss via forming more input impedance (*Z*_in_) instead of reflecting to the free space. Z is evaluated based on Eq. (3) [50]:

3 $$Z=\left|\frac{{Z}_{in}}{{Z}_{0}}\right|=\sqrt{{\mu }_{r}/{\varepsilon }_{r}}$$

where *Z*_in_ is incidence impedance, and *Z*_0_ is the impedance of air. When |*Z*_in_/*Z*_0_|= 1, the impedance of the absorber is absolutely matched with the free space, and the microwave could be penetrated totally without being reflected. As displayed in Fig. S17, the best impedance matching of air@G-Fe/C nanospheres could be achieved at 13.1, 6.6, 10.5, and 16.0 GHz, respectively. Simultaneously, the reflection loss could achieve the peak intension at the corresponding frequency to consume microwave rather than reflecting the contacting area between the absorbing material and air. Generally, the degree of |*Z*_in_/*Z*_0_| in the scope of 0.8—1.2 indicates that the attenuation feature and impedance matching ability can hold an equilibrium. In Fig. 5i-l, Z value plots of air@G-Fe/C nanospheres are displayed as a function of frequency and thickness. As revealed in Fig. 5i-j, T180 and T190 do not present an effectively damping capacity, as there is only an impedance matching degree of 13.5% and 14.2% in the scope of 0.8–1.2. The |*Z*_in_/*Z*_0_| degrees of T200 and T210 enhance to 18.1.0% and 16.7%, respectively (Fig. 5k-l). This result manifests that the impedance matching characteristic has been improved triumphantly with the gradient structure design, deriving from the optimized conductivity attenuation ability. Moreover, the Z parameters of air@G-Fe/C nanospheres are figured out further with five specific thicknesses (*d* = 1–5 mm) in Fig. S18. Similarly, T180 and T190 could not acquire available impedance matching properties; nevertheless, T200 and T210 have superior impedance matching properties, demonstrating the improved electromagnetic wave absorption characteristic of the optimized T200 and T210 is primarily benefited from the excellent impedance matching, yet. Therefore, the multiple reflections caused by gradually increasing conductivity Fe NPS is the dominative factor for the excellent EMA capacity of air@G-Fe/C nanospheres, which can also be enhanced availably via the gradient distribution of other conductive NPs, line well with our initial design target.

### Effect of Annealing Temperature on Air@G-Fe/C Sphere Interior Interface and Microwave Absorbing Performance

**Fig. 6:**
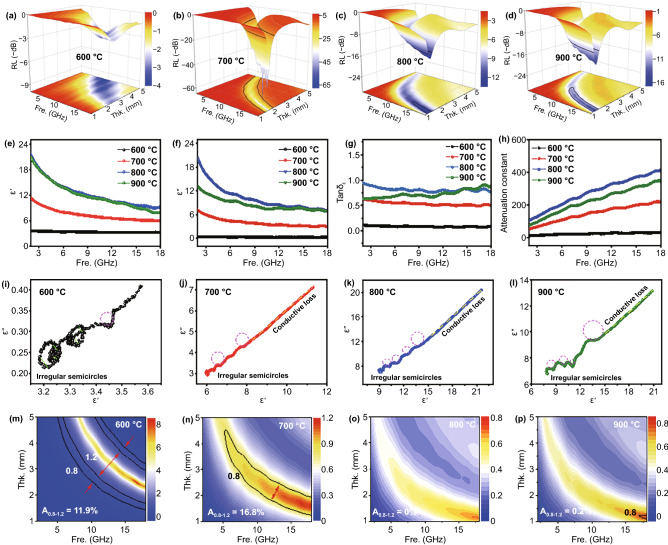
RL and electromagnetic parameters of air@G-Fe/C nanospheres with different carbonization temperatures. The 3D RL of **a** T600, **b** T700, **c** T800, and **d** T900. **e** ε′, **f** ε′′, **g** The tan*δε* plots (tan*δε* = *ε''/ε'*) **h** α. Cole–Cole plots of **i** T600, **j** T700, **k** T800, and (**l**) T900. Calculated |Z_in_/Z_0_| maps of **m** T180, **n** T190, **o** T200, and **p** T210

The RL value and broadened frequency responding ability of air@G-Fe/C nanospheres could be regulated by regulating the dielectric parameters. Selected samples were heated at 600, 700, 800, and 900 °C, which were denoted as T600, T700, T800, and T900, respectively. Figure 6a-d presents the 3D RL maps of different nanospheres, and the peak *RL* of T600 just achieves − 3.4 dB, which is only ~ 6 percent of T700 (Fig. S19a-b). When the pyrolysis temperature is increased to 800 and 900 ℃, the *RL* ability and broadened frequency responding performance show an opposite trend, with the peak RL values decreasing to − 13.7 dB (T800, 1.3 mm) and − 14.2 dB (T900, 1.35 mm), and the EAB performances reduce to 3.0 GHz (15.0–18 GHz) and 5.1 GHz (12.9–18 GHz), respectively (Fig. S19c-d). That is to say, the EMA performance can be efficiently regulated by the annealing process. The components, interfaces, and impedance matching are further analyzed to figure out the adjusting mechanism of the annealing process. As presented in Fig. S20a, the iron phase is transformed from Fe_3_O_4_ to Fe and then to FeC_3_ consecutively with the increase in heating temperature. Consistent with our outlook, the iron concentration in all air@G-Fe/C samples is similar (~ 70 wt%) (Fig. S20b-c). As shown in Fig. S21, samples handled by 900 ℃ display a stronger intensity of Fe(OH)O bond, attributing to the bigger grain size by high temperature. Besides, due to the higher induced graphitization phenomenon caused by uniformly dispersed Fe [33], the composition of Fe_3_C in 900 ℃ increases to 55%. Samples carbonized under 600 ℃ demonstrate higher Fe_3_O_4_ contents, consistent with the result in Fig. S19a. Magnetic hysteresis loops revealed in Fig. S19d demonstrate that Ms is in the order of T800 > T700 > T900 > T600, which is inconsistent with the rule of electromagnetic wave absorption performance representing the negligible role of magnetic loss. µ′ and µ″ of different air@G-Fe/C nanospheres are ~ 1.1 and ~ 0.05, respectively, and the relevant tanδ_m_ is only 0.05 (Fig. S22a-c), smaller than the dielectric loss tangent (0.5 ~ 1.0, Fig. 6g). Moreover, the eddy current coefficient (*C*_*0*_) reduces and keeps stability from 6.0 GHz, revealing that the magnetic loss of iron NPs could be neglected as well (Fig. S21d).

The temperature dependence of carbon demonstrated via Raman spectra characterization reveals that the intensity of *I*_G_/*I*_D_ attenuates weakly from 0.73 to 0.58 (Fig. S20e) with the increasing of annealing temperature, boosting the overall carrier mobility. As seen in Fig. 6e, a, larger ε' intensity can be gained in T800 and T900. In contrast to T600, the ε'' of T700, T800, and T900 sharply dropped in the range of 2–7.5 GHz and remained stable in the rest of scope (Fig. 6f), proving that conductive loss plays a dominant effect in the dielectric loss. An identical verdict can be acquired by the Cole–Cole plot, as shown in Fig. 6i-l. There are three dielectric relaxation peaks in T600, indicating a prominent multi-polarization loss process (Fig. 6i). Nevertheless, there is no obvious semicircle in T700 and T800, demonstrating that a weaker polarization relaxation will form with the increase of heating temperature (Fig. 6j-k). Continuous improvement of the annealing temperature will facilitate the transformation of Fe to FeC_3_, leading to more interfaces and defects, and disruption the continuity carbon conductive network (Figs. S20a, e, and S21). The inherent dielectric loss property could be elucidated via α. However, the α is in the order of T800 > T900 > T700 > T600, which is inconsistent with the role of electromagnetic wave absorption performance, representing the indecisive role of dielectric loss in microwave loss as well (Fig. 6h).

An ideal electromagnetic wave-absorbing material should synchronously hold superior microwave loss capacity and eminent impedance matching performance. Thus, the outstanding EMA performance and lower α characteristic of gradient-structured T700 might be attributed to inferior impedance mismatching traits. As shown in Fig. 6m-p, the calculated impedance matching degree (|*Z*_in_/*Z*_0_|) of T800 and T900 is only 0% and 0.2%, much lower than that of T600 and T700. Meanwhile, the T700 reveals a significantly superior impedance matching performance at all calculated thicknesses (1–5 mm, Fig. S23). Therefore, the effect of annealing temperature on dielectric loss and magnetic loss should be neglected, and the dominating role of air@G-Fe/C nanospheres in the EMA ability comes from the regulation of impedance mismatching, which is modulated by the appropriate conductivity property caused by the gradient dispersion of Fe NPs (Figs. S24 and S25c-d).

The correlative microwave-absorbing mechanism is further explored according to the analysis above (Fig. S26). The microporous and mesoporous G-Fe/carbon shell is deemed to be composed of continuous graphitized carbon and a lot of irregular Fe NPs. Commonly, these Fe NP-based areas are discontinuous, but determine the integrated electronic migration of carbon shell [51]. The addition of Fe NPs reveals the improvement of electric conductivity for air@G-Fe/C nanoball, which is benefitted for the improvement in both impedance matching and dielectric attenuation. Besides, it can be simply adjusted by the distribution of Fe NPs or the graphitization degree of carbon shell, for some adjacent Fe NPs and graphitization zones will integrate with each other and form more prominent graphitization zones [18]. The well balance between the dispersion of conductive particles and graphitization degree of carbon for the optimized T200 and T210 composites lead to a strong absorption feature and broad-frequency EMA property. The primary optimized factors might result from the following ways. Firstly, porous carbon shells, anchored with Fe NPs, construct micro-current conductive networks, which is extremely important for multiple reflections and loss of microwave [4]. In addition, the improvement of interface polarization was induced via charges assembling at the air/Fe/graphitized carbon interface, and the dipole polarization evoked via functional group and defect result in the outstanding dielectric damping of air@G-Fe/C nanoball [52]. Moreover, magnetic loss also displays a weak function in the absorption and attenuation of electromagnetic waves. Simultaneously, due to the designability dispersion of conductive particles and tunable graphitization degree of carbon, impedance matching character and interfacial polarization are adjustable in the air@G-Fe/C absorber. Based on the competitive reaction and appropriate process optimization, other metal ions (i.e., Cu, Ag, Co, Ni) can be used to adjust the EMA feature for the hollow carbon shell as well.

## Conclusions

In summary, we proposed a new tactics to fabricate a durable hollow carbon sphere microwave absorber with gradient-distributed micro-conductive structure and tunable microwave-absorbing performance. The combination of gradient-dispersed ultra-small Fe nanoparticles and mesoporous graphitized carbon shell gradually increases micro-current conductive networks at the atomic scale from surface to inside, resulting in outstanding comprehensive microwave absorption properties (i.e., high RL and wide EAB). It demonstrates that a balance between the dispersion of conductive particles and the graphitization degree of carbon can be adjusted easily via the solvothermal and annealing process. In addition, the novel strategy could be effectively extended to other metal ions or alloys, to implement tunable microwave absorption ability. The optimal RL value achieves -62.7 dB and EAB achieves 6.4 dB with only 2.1 mm thickness. Compared with traditional gradient distribution/multilayer structures, the air@G-Fe/C nano-ball presented in this work reveals obvious advantages, such as strong RL value, broadband absorption, lightweight, excellent anti-fading character, and satisfactory manufacturing precision, fitting well with the requirements of microwave absorption materials. This way to fabricating gradient distribution structures as a microwave absorber initiates a new insight on the design and modulation of electromagnetic wave absorption materials in practical use.

### Supplementary Information

Below is the link to the electronic supplementary material.

Supplementary file1 (PDF 1617 kb)
