# Supplementary material for: Hollow Gradient-Structured Iron-Anchored Carbon Nanospheres for Enhanced Electromagnetic Wave Absorption
Source: Nanomicro Lett. 2022 Dec 6;15:7. doi: 10.1007/s40820-022-00963-w (PMC9727008; doi:10.1007/s40820-022-00963-w)
Supplement: Supplementary file 1 — Supplementary file1 (PDF 1617 kb) [file 40820_2022_963_MOESM1_ESM.pdf]

Supporting Information for

Hollow Gradient-Structured Iron Anchored Carbon Nanospheres for Enhanced Electromagnetic Wave Absorption

Cao Wu[1, †], Jing Wang[2, 3, †], Xiaohang Zhang[2], Lixing Kang[1, 4, *], Xun Cao[5], Yongyi Zhang[1, 3, 4, *], Yutao Niu[1, 3, 4], Yingying Yu[1, 9], Huili Fu[1, 3, 4], Zongjie Shen[1], Kunjie Wu[1, 3], Zhenzhong Yong[1, 3, 4], Jingyun Zou[6], Bin Wang[3], Zhou Chen[7], Zhengpeng Yang[8], and Qingwen Li[1, 4, *]

[1] Key Laboratory of Multifunctional Nanomaterials and Smart Systems, Advanced Materials Division, Suzhou Institute of Nano-Tech and Nano-Bionics, Chinese Academy of Sciences, Suzhou 215123, Jiangsu, P. R. China

[2]School of Science, Nanchang Institute of Technology, Nanchang 330099, Jiangxi, P. R. China

[3]Division of Nanomaterials and Jiangxi Key Lab of Carbonene Materials, Jiangxi Institute of Nanotechnology, Nanchang 330200, Jiangxi, P. R. China

[4]School of Nano-Tech and Nano-Bionics, University of Science and Technology of China, Hefei 230026, Anhui, P. R. China

[5]School of Materials Science and Engineering, Nanyang Technological University, 50 Nanyang Avenue, 639798, Singapore

[6]Jiangsu Key Laboratory of Micro and Nano Heat Fluid Flow Technology and Energy Application, School of Physical Science and Technology, Suzhou University of Science and Technology, Suzhou 215009, P. R. China

[7]School of Mechanical and Power Engineering, Nanjing Tech University, Nanjing 211800, P. R. China

[8]Henan Key Laboratory of Materials on Deep-Earth Engineering, School of Materials Science and Engineering, Henan Polytechnic University, Jiaozuo, 454003, P. R. China

[9]College of Safety Science and Engineering, Xi'an University of Science and Technology, Xi'an 710054, P. R. China

[†]Cao Wu and Jing Wang contributed equally to this work.

*Corresponding authors. E-mail: lxkang2013@sinano.ac.cn (L. Kang); yyzhang2011@sinano.ac.cn (Y. Zhang); qwli2007@sinano.ac.cn (Q. Li)

S1 Electromagnetic Parameters Calculation

The coaxial-line method was used to test the S parameters (means S11, S12, S21 and S22). The corresponding electromagnetic parameters (ε′, ε″, μ′, μ″) could be figured out by software which is installed on the Agilent PNA. Reflection loss (RL) values were evaluated by their complex permittivity and permeability via following formula [S1]:

$$Z_{in} = Z_o(\mu_r/\varepsilon_r)^{\frac{1}{2}}\tanh\left[j(2\pi f d(\mu_r\varepsilon_r)^{\frac{1}{2}}/c)\right] \quad \text{(S1)}$$

$$\mathrm{RL(dB)} = 20\log\left|\frac{Z_{in}-Z_0}{Z_{in}+Z_0}\right| \quad \text{(S2)}$$

where Z and Z_0 are incidence impedance and impedance of air (377Ω) [S2], respectively, *c* and *f* are theoretical velocity and frequency of input electromagnetic waves, and *d* is thickness of electromagnetic wave absorber. The attenuation constant (α) could be assessed by transmission line theory, and the corresponding calculation formula is as follows: [S3, S4]

$$\alpha = \frac{\sqrt{2}\pi f}{c}\sqrt{(\mu''\varepsilon'' - \mu'\varepsilon'') + \sqrt{(\mu''\varepsilon'' - \mu'\varepsilon')^2 + (\mu'\varepsilon'' - \mu''\varepsilon')^2}} \tag{S3}$$

S2 Supplementary Tables and Figures

Table S1 The comparison of the wave absorption performance for air@G-Fe/C nanospheres and SiO_2@G-Fe/C counterpart at the scope of 2.0-18.0 GHz

Materials	RL_{min} (-dB)	EAB (GHz)	QBW (GHz)	Thickness [(a)] (mm)	Density (mg cm^{-3})
T200 (This work)	55.97	6.2	14	2.0	83
T210 (This work)	62.7	6.4	13.85	2.1	83
SiO_2@G-Fe/C [(b)]	22.4	6.2	13.8	2.0	2180

Note:

[(a)]The thickness in the peak RL value;

[(b)]The solvothermal temperature of solid counterpart (SiO_2@G-Fe/C) was 210 °C

Table S2 The calculated concentration of Fe(OH)O, Fe_3C, Fe_3O_4 and O_2/Fe/Cu

Calculated concentration	T180	T190	T200	T210
O1s，Fe(OH)O/% [(a)]	0.462	0.759	0.351	0.247
Fe 2p，Fe_3C/% [(a)]	0.43	0.45	0.54	0.58
Fe 2p，Fe_3O_4/% [(a)]	0.2	0.39	0.23	0.28
Fe 2p，O_2/Fe/Cu/% [(a)]	0.125	0.224	0.244	0.285

Note:

[(a)] Concentration was calculated from X-ray photoelectron spectroscopy (XPS)

Table S3 The surface area and pore size analyzer analysis of air@G-Fe/C nanoballs

Samples	T180	T190	T200	T210
BET surface （$m^2 \cdot g^{-1}$)	281.74	229	156.52	83.054
Mean pore diameter（nm）	6.35	6.91	5.75	5.05
Dpeak (nm)	1. 4	1.9	2.0	1.8

Table S4 The comparison of the wave absorption performance of different gradient distributions/multi-layer materials at the scope of 2.0-18.0 GHz

Structure and Preparation method	RL_{min} (-dB)	EAB (GHz)	QBW (GHz)	Thickness [(a)] (mm)	Precision (nm)	Density (mg cm^{-3})
T200 (This work)	55.97	6.2	14	2.0	~20	83
T210 (This work)	62.7	6.4	13.85	2.1	~20	83
FeSiAl@Al_2O_3@SiO_2 Core–shell (Plasma) [S5]	46.29	7.33	15	2.5	50	~3175[(b)]
ZnO-Al_2O_3-CNF fiber (ALD) [S6]	58.5	6	12.8	1.8	15	~1600[(b)]
CNTs/SiO_2 composites	9.5	3.4	3.4	5	800	~1558[(b)]

(Hot-pressed sintering) [S7]						
MXene/polymer films (Tape casting process) [S8]	26.1	1.44	4.2	7	138	1000[(b)]
RGO/CNC/CNF/M-NPs hierarchical aerogel (Hydrothermal-Freeze-drying-CVD) [S9]	71.5	4.5	14	2.95	500-3000	— [(c)]
TiO2@Co/C@Co/Ni multilayered microtubes. Electrospinning [S10]	53.99	6.0	~14	2.0	~100	— [(c)]
Hollow Fe@Carbon Templates method [S11] dual-shells	54.4	8.1	— [(c)]	4.5	— [(c)]	— [(c)]
Gradient Hierarchical Porous (lotus leaf) [S12]	50.1	5.8	~13	2.4	300-5000	— [(c)]

Note:

[(a)]The thickness in the peak RL value

[(b)]The dates calculated corresponding to the structure and component

[(c)]data are not available

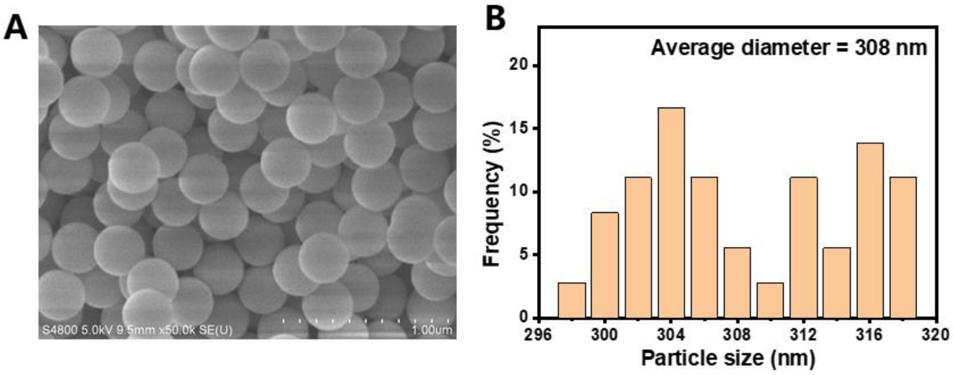

Fig. S1 (**A**) SEM images of colloidal SiO_2 nanoballs, (**B**) and their statistics results of particle size distribution

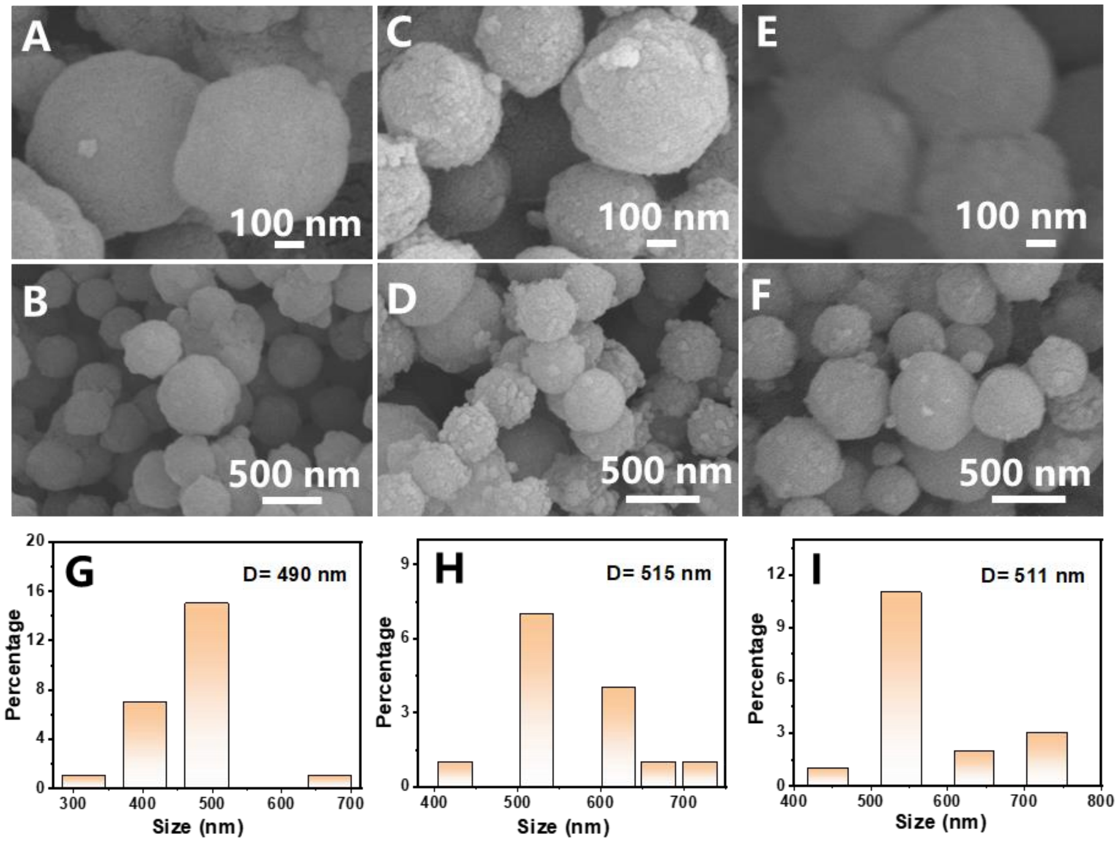

Fig. S2 SEM images of SiO_2@G-Fe_3O_4/C precursor obtained from solvothermal temperature of (**A, B**)180 ℃, (**C, D**)190 ℃and (**E, F**)200 ℃; The size distributions of SiO_2@G-Fe_3O_4/C precursor calculated from (**G**)180 ℃, (**H**)190 ℃and (**I**)200 ℃, respectively

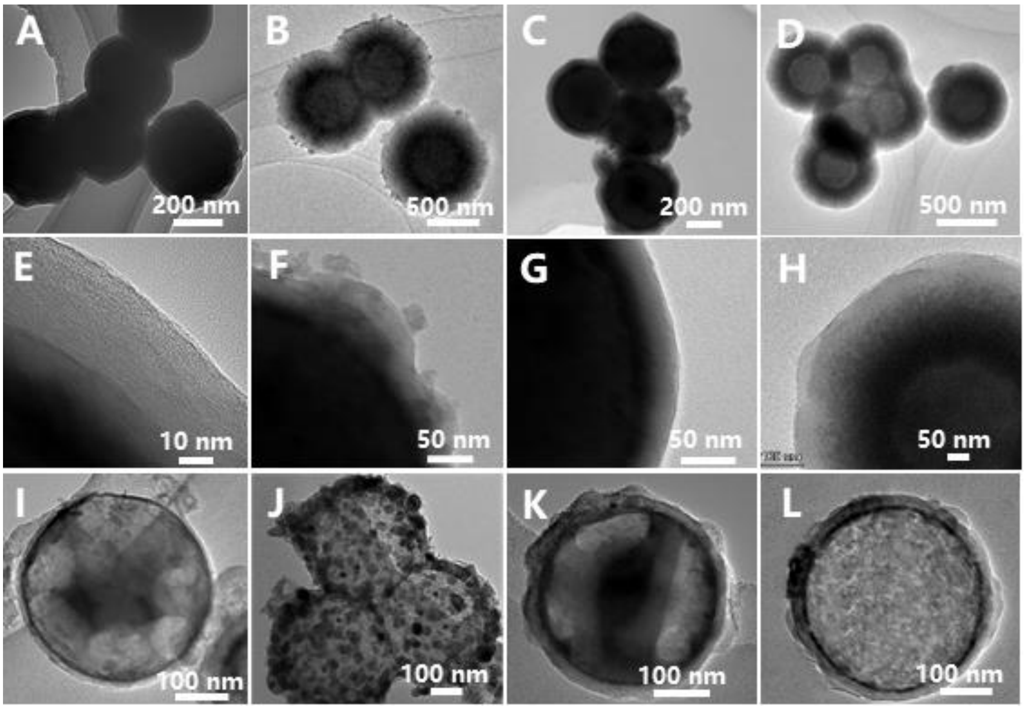

Fig. S3 TEM of SiO_2@G-Fe_3O_4/C precursor prepared by solvothermal temperature of (**A, E**)180 ℃, (**B, F**)190 ℃, (**C, G**)200 ℃and (**D, H**)210 ℃. The corresponding air@G-Fe/C nanoballs products of (**I**)T180, (**J**)T190, (**K**)T200 and (**L**)T210

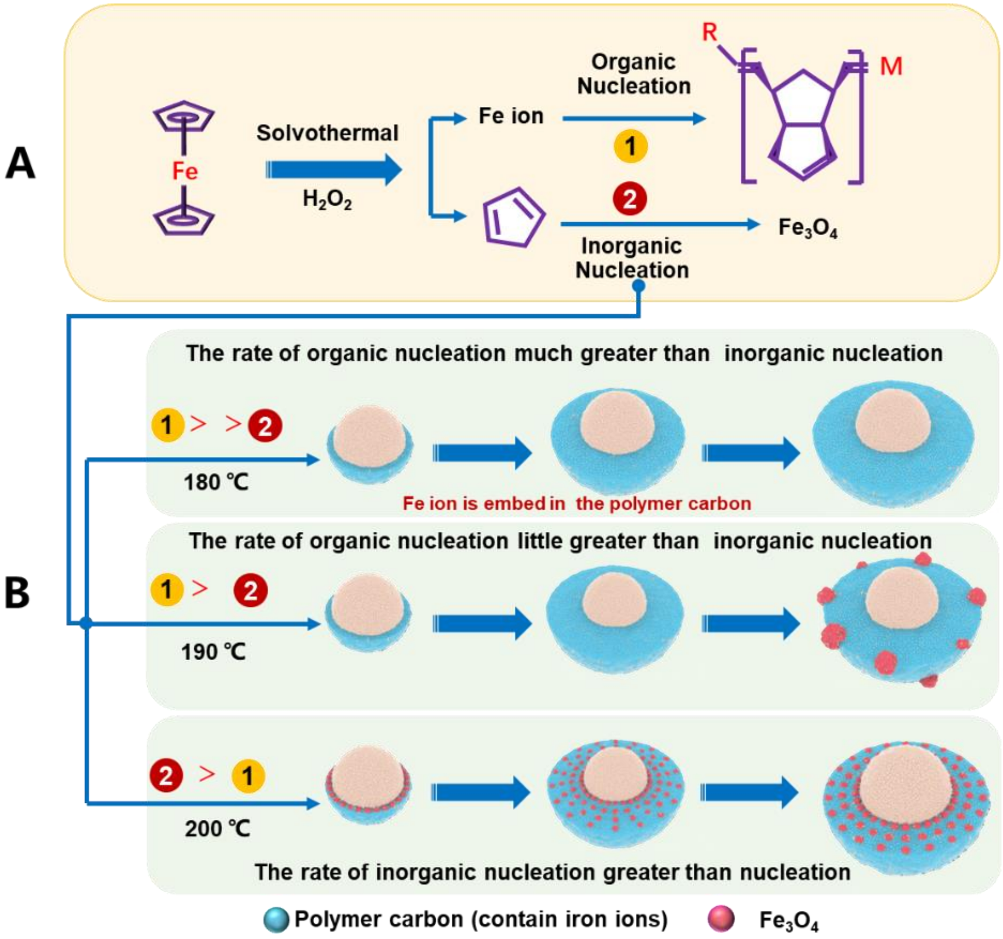

Fig. S4 Schematic of the inorganic-organic competitive coating strategy in solvothermal process. (**A**) The ferrocene is gradually hydrolyzed into Fe ions and cyclopentadiene, and then Fe ions are further hydrolyzed into hydrated Fe_3O_4 (inorganic nucleation), and cyclopentadiene are oxidized and polymerized into amorphous carbonaceous species (organic nucleation), (**B**) Schematic diagram for nucleation rate between iron oxides and amorphous carbonaceous species and model diagram of competitive coating process by solvothermal reaction temperature of 180, 190, and 200 °C

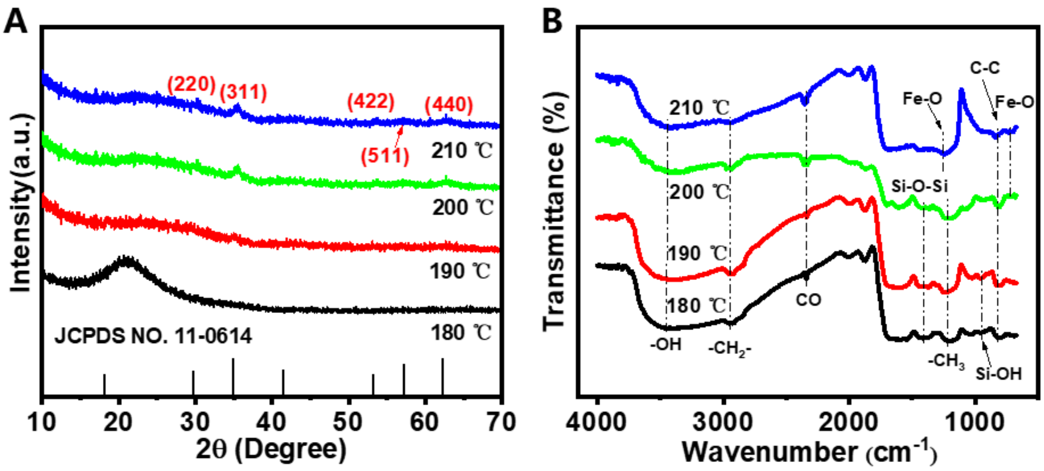

Fig. S5 (**A**) XRD and (**B**) FT-IR spectroscopy of SiO_2@G-Fe_3O_4/C precursor with different solvent thermal temperature treatment

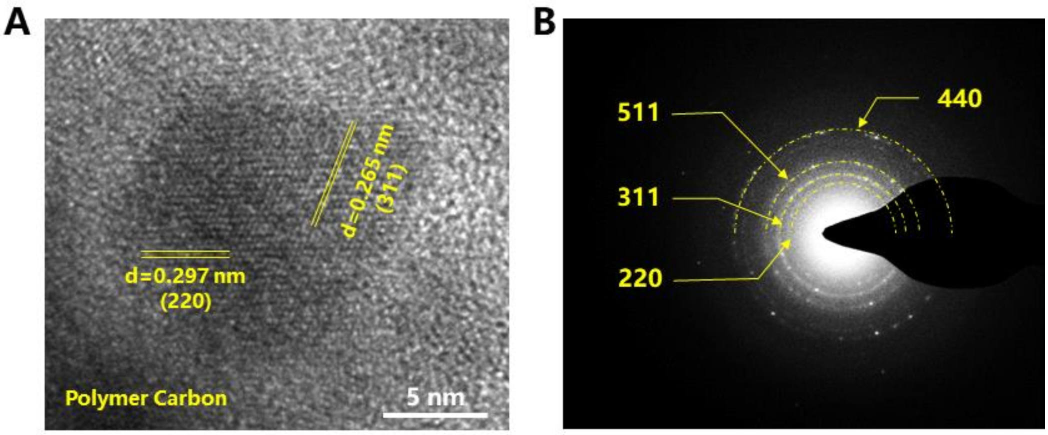

Fig. S6 (**A**) HRTEM image and (**B**) selective area electronic diffraction (SAED) pattern of SiO_2@G-Fe_3O_4/C precursor

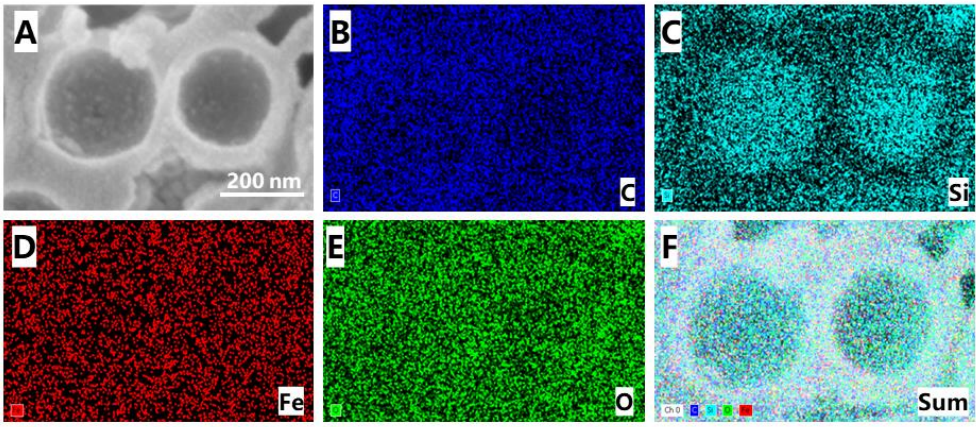

Fig. S7 (**A**) SEM and (**B-F**) the corresponding EDS mapping of SiO_2@G-Fe_3O_4/C precursor prepared by FIB

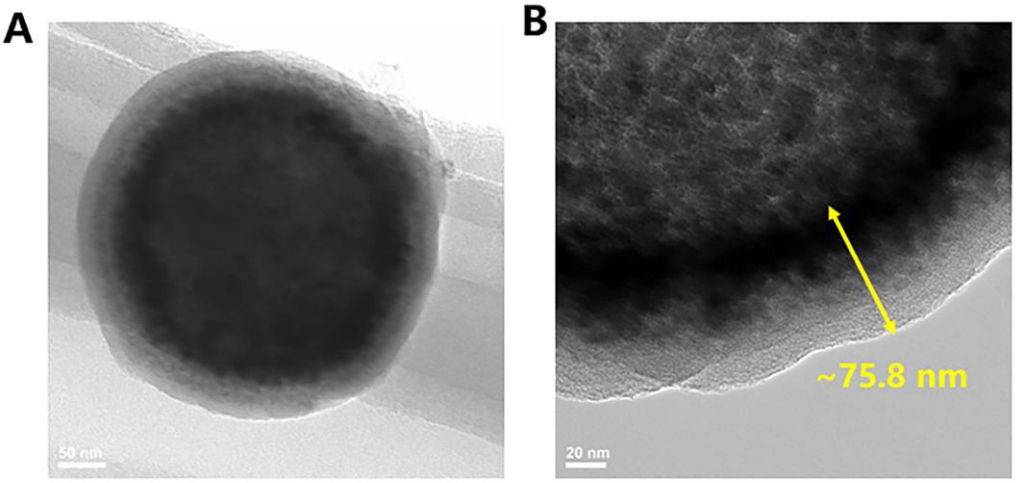

Fig. S8 (**A**) TEM and (**B**) the magnification images of graded distributed Fe/C nanospheres

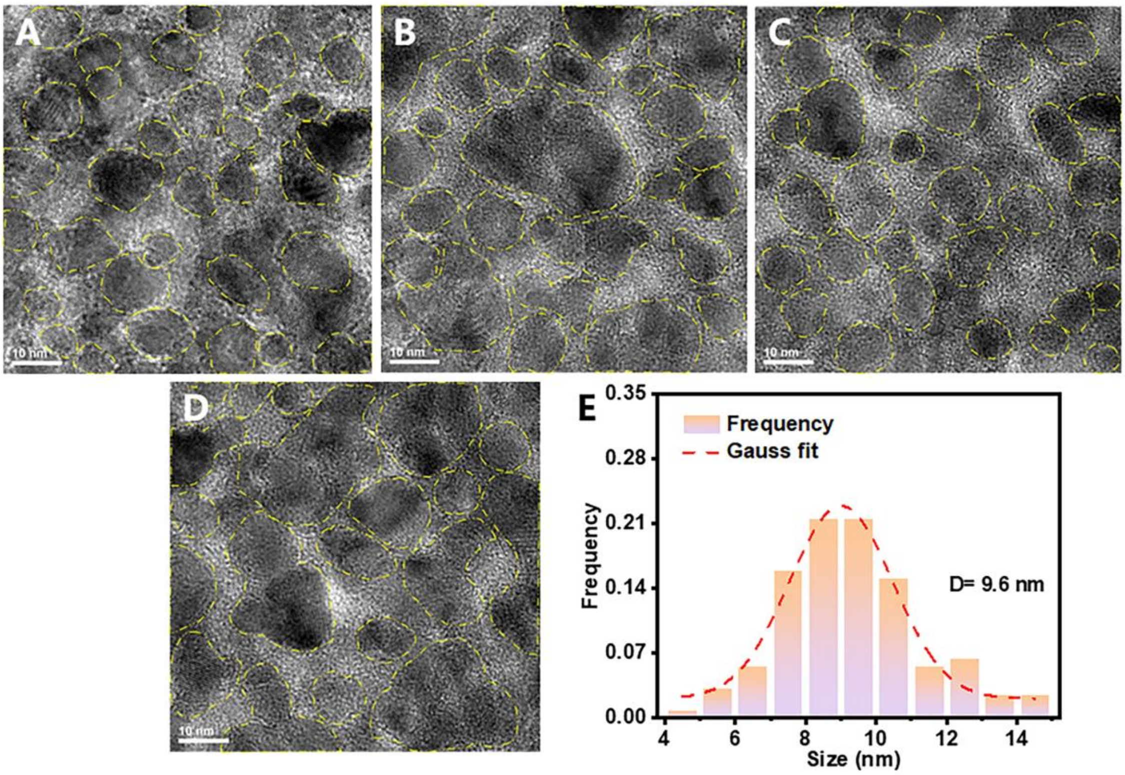

Fig. S9 (**A-D**) HRTEM images of graded distributed Fe/C nanospheres, and (**E**) the corresponding size distributions of Fe nanoparticles calculated from (**A-D**)

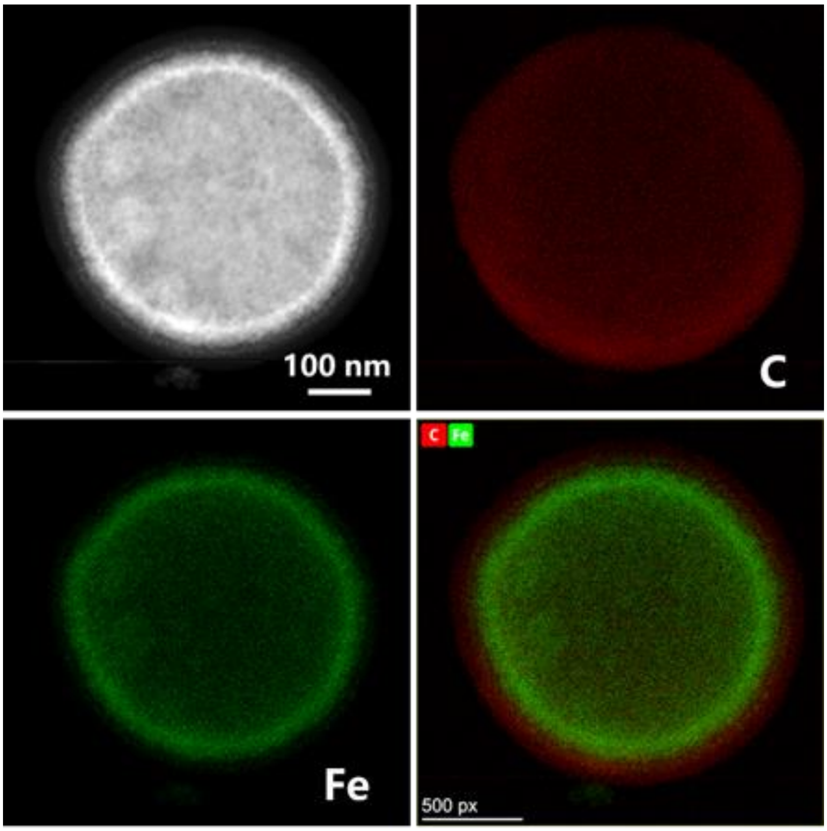

Fig. S10 HAADF image and elemental mapping images of T210

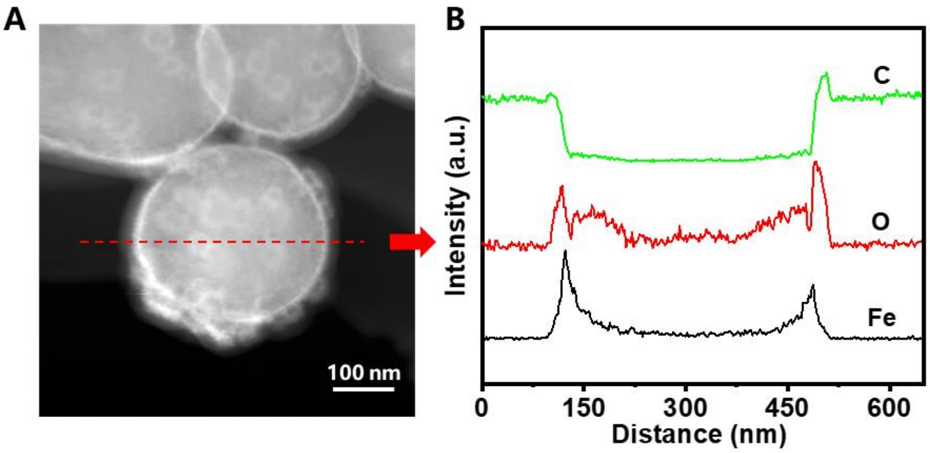

Fig. S11 (**A**) HAADF image and (**B**) the corresponding EDS line scan of air@G-Fe/C-200 nanoballs

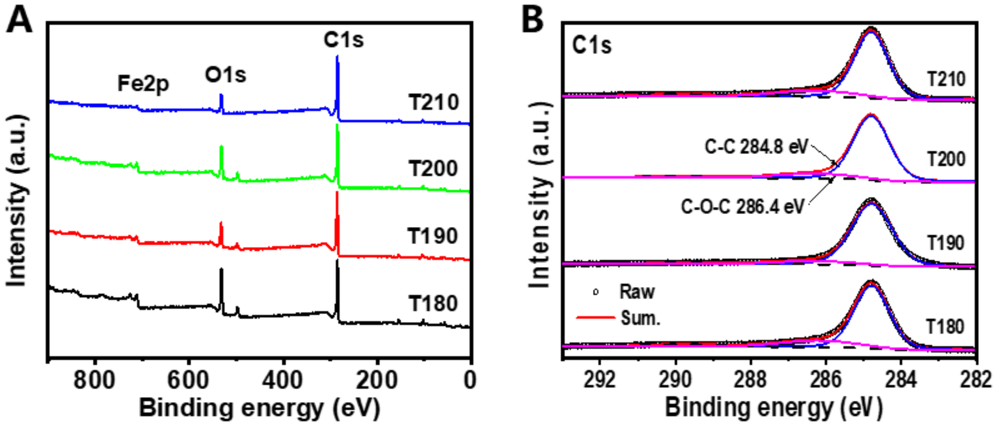

Fig. S12 (**A**) wide-scan survey of XPS spectra and (**B**) high-resolution XPS signals of C 1s

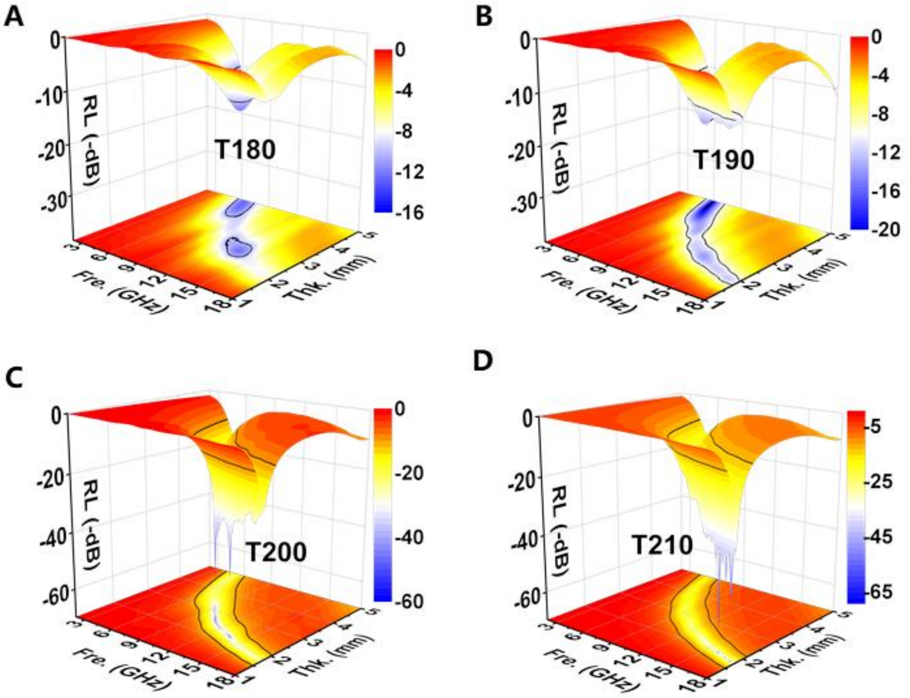

Fig. S13 3D reflection loss (RL) values of (**A**) T180, (**B**) T190, (**C**) T200, and (**D**) T210 with different thickness and frequency

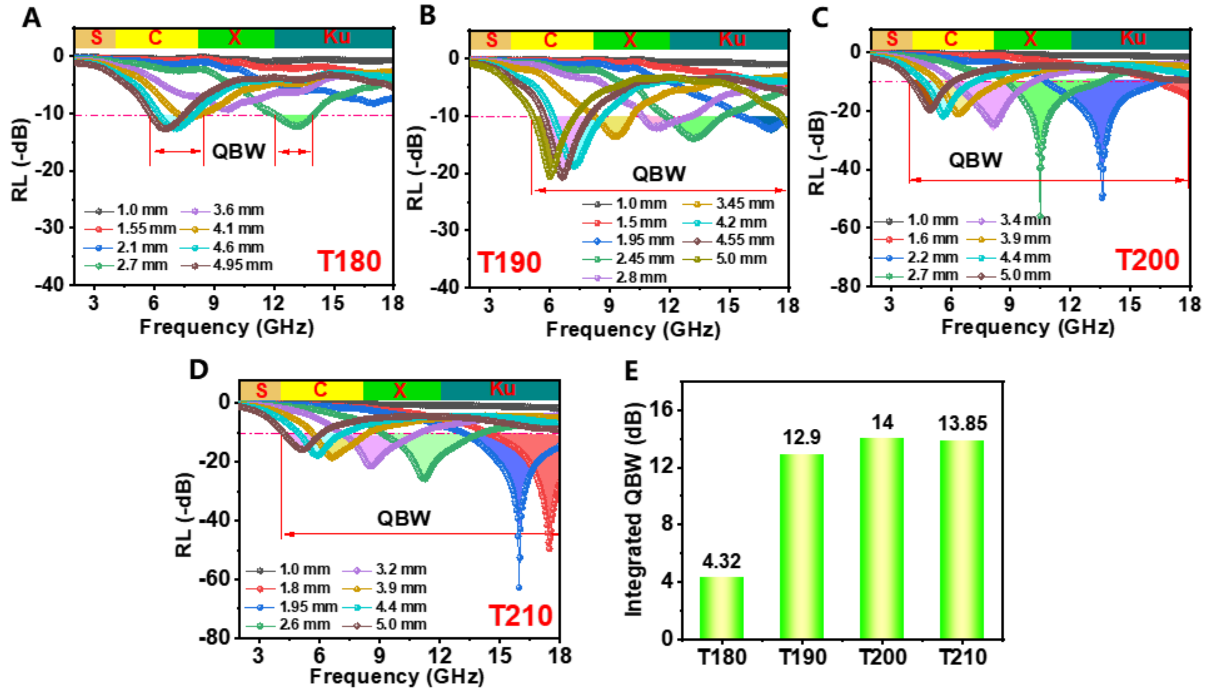

Fig. S14 The dependence of RL values on the thickness of (**A**) T180, (**B**) T190, (**C**) T200, and (**D**) T210 of hollow air@G-Fe/C-200 nanoballs. (**E**) The curves of integrated QBW

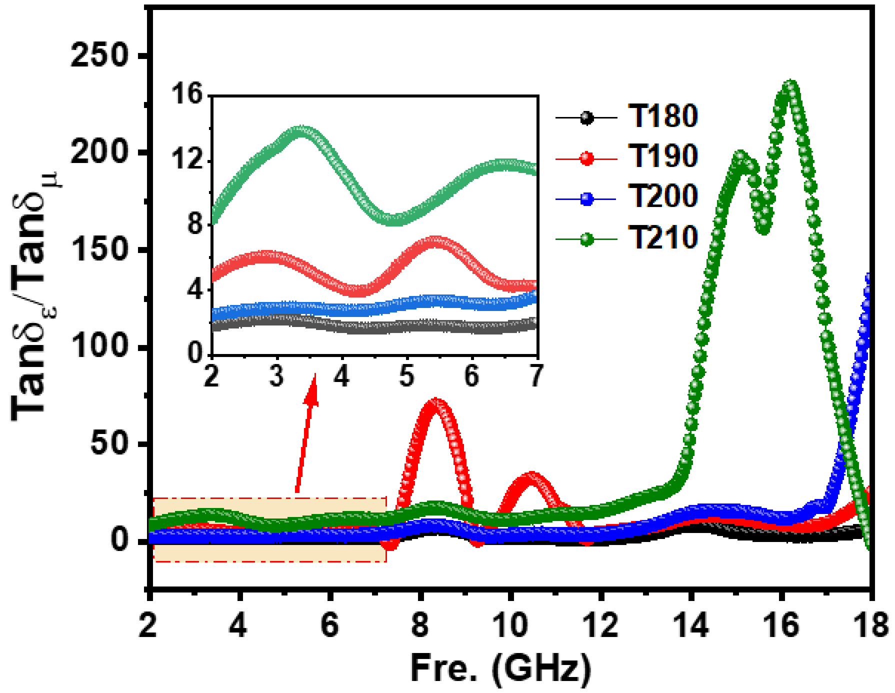

Fig. S15 The absolute value of $Tan\delta_\varepsilon/Tan\delta_\mu$curves in the range of 2 – 18 GHz, Insert the partial enlargement of $Tan\delta_\varepsilon/Tan\delta_\mu$curves

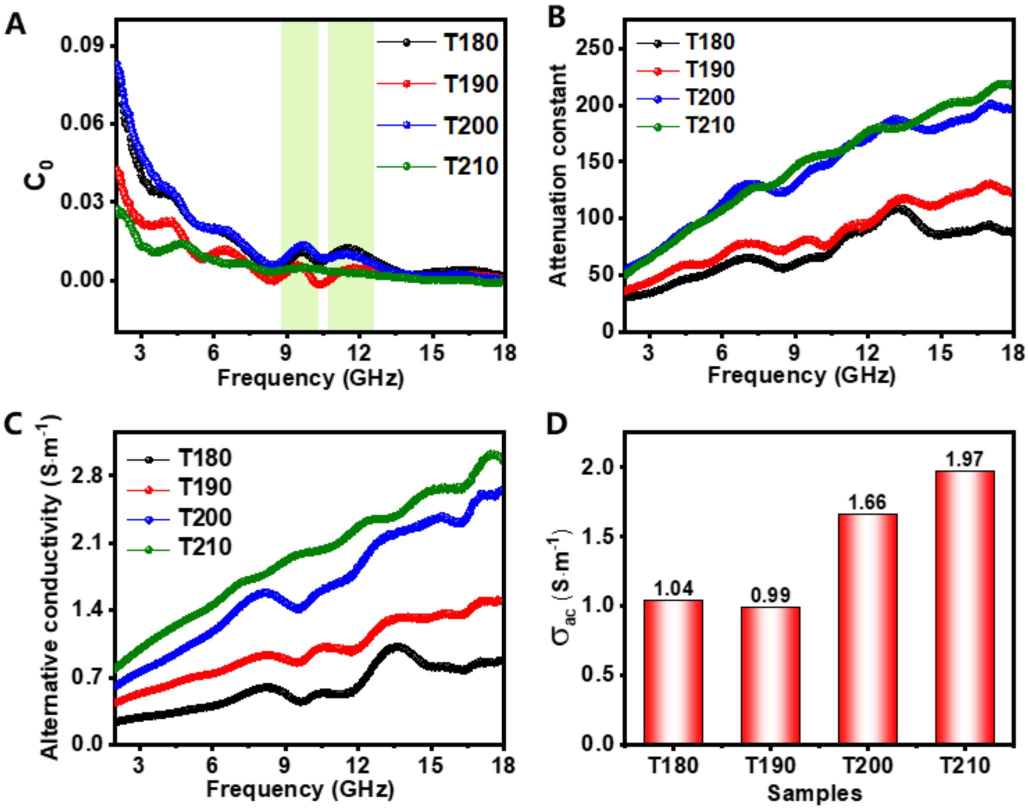

Fig. S16 (**A**)The eddy current values ($C_0 = \mu''(\mu')^{-2}f^{-1}$), (**B**)Attenuation constant, (**C**)Alternative conductivity, and the corresponding average conductivity (σac, **D**) of different samples

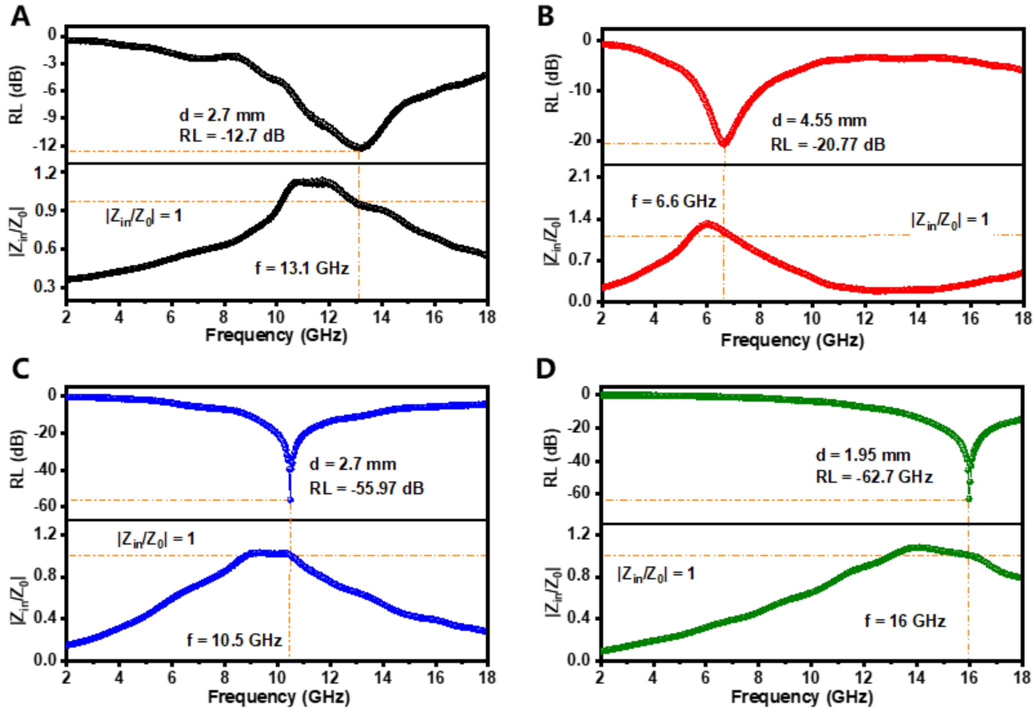

Fig. S17 The impedance matching degree $|Z_{in}/Z_0|$ values of (**A**) T180, (**B**) T190, (**C**) T200, and (**D**) T210

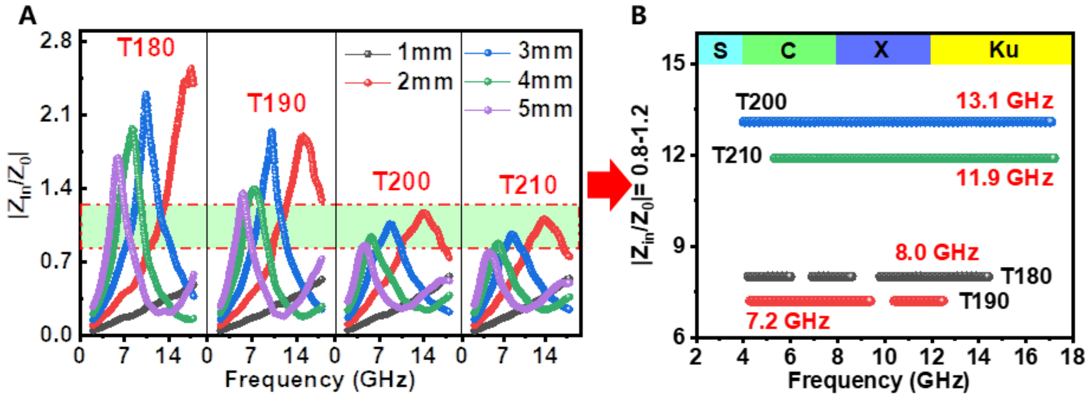

Fig. S18 (**A**) The $|Z_{in}/Z_0|$ values with thicknesses of 1 – 5 mm, and (**B**) the values between 0.8-1.2 in the range of 2-18 GHz

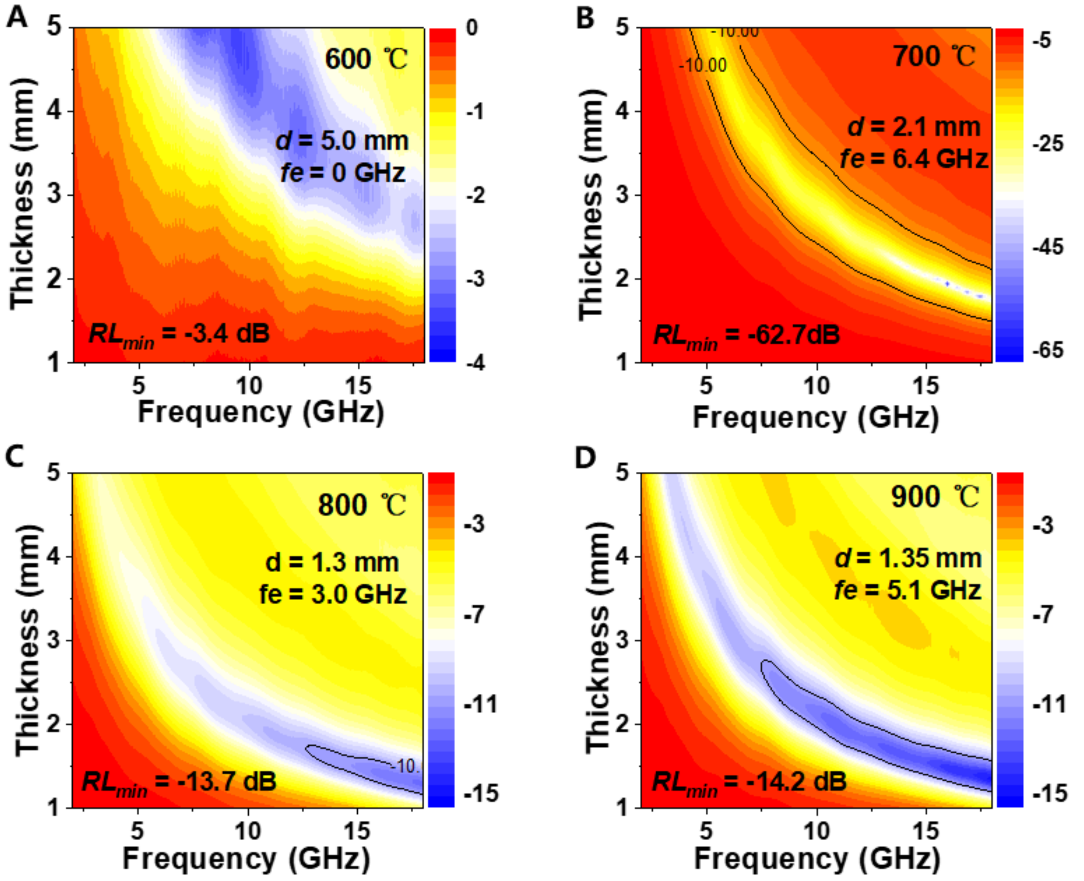

Fig. S19 The reflection loss (RL) values of (**A**) T600, (**B**) T700, (**C**) T800, and (**D**) T900 with different thickness and frequency

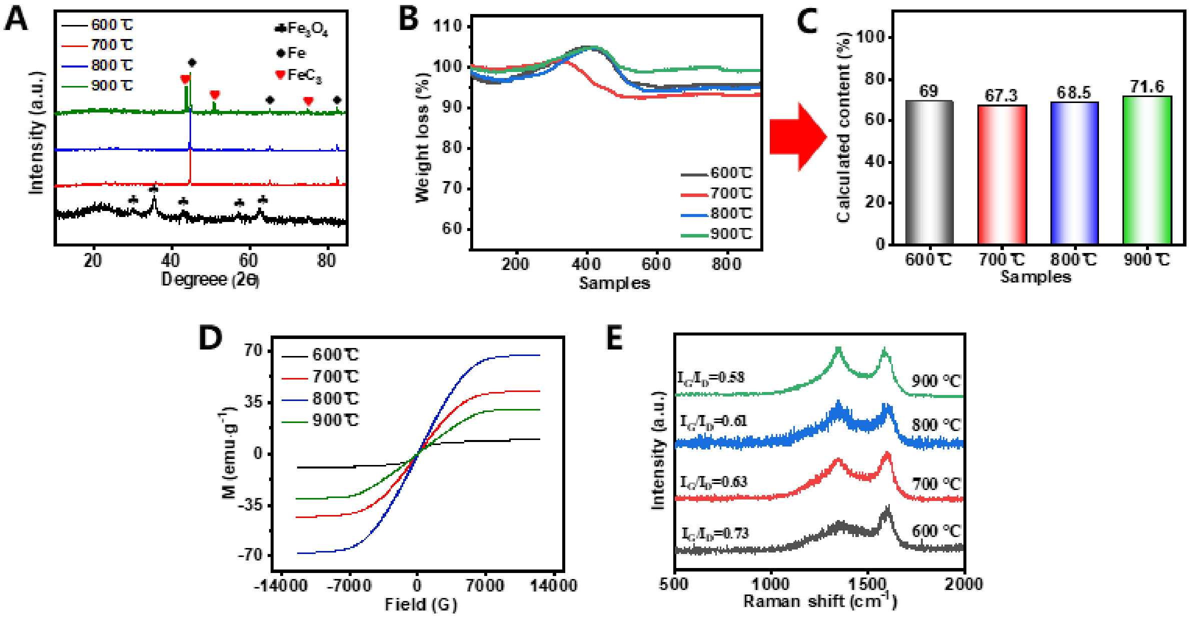

Fig. S20 Compositional characterization of air@G-Fe/C nanospheres for different annealing temperature. (**A**) XRD patterns, (**B**) TGA curves, and (**C**) the corresponding calculated content of iron, (**D**) Magnetic hysteresis loops at 298 K, and (**E**) Raman spectrum

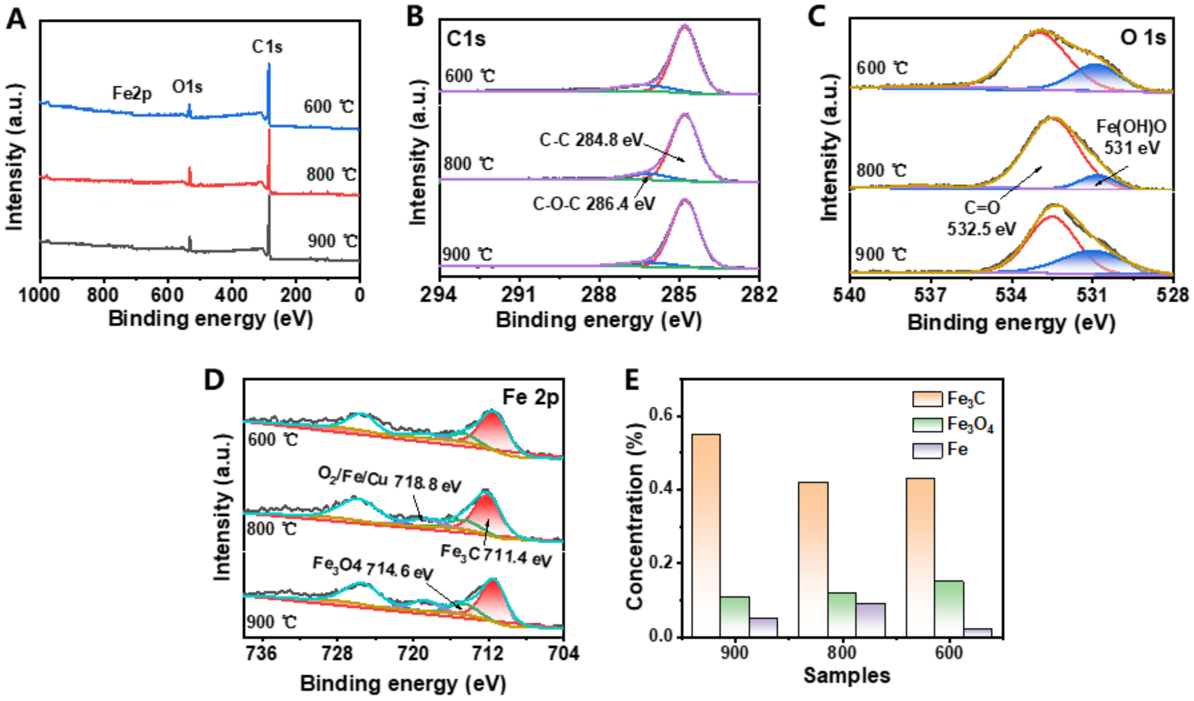

Fig. S21 Compositional characterization of air@G-Fe/C nanospheres by XPS. (**A**) wide-scan survey of XPS spectra, (**B**) high-resolution XPS signals of C 1s, (**C**) O $_{1s}$, (**D**) Fe $_{2p}$, and (**E**) the corresponding calculated concentration of Fe_3C, Fe_3O_4 and Fe

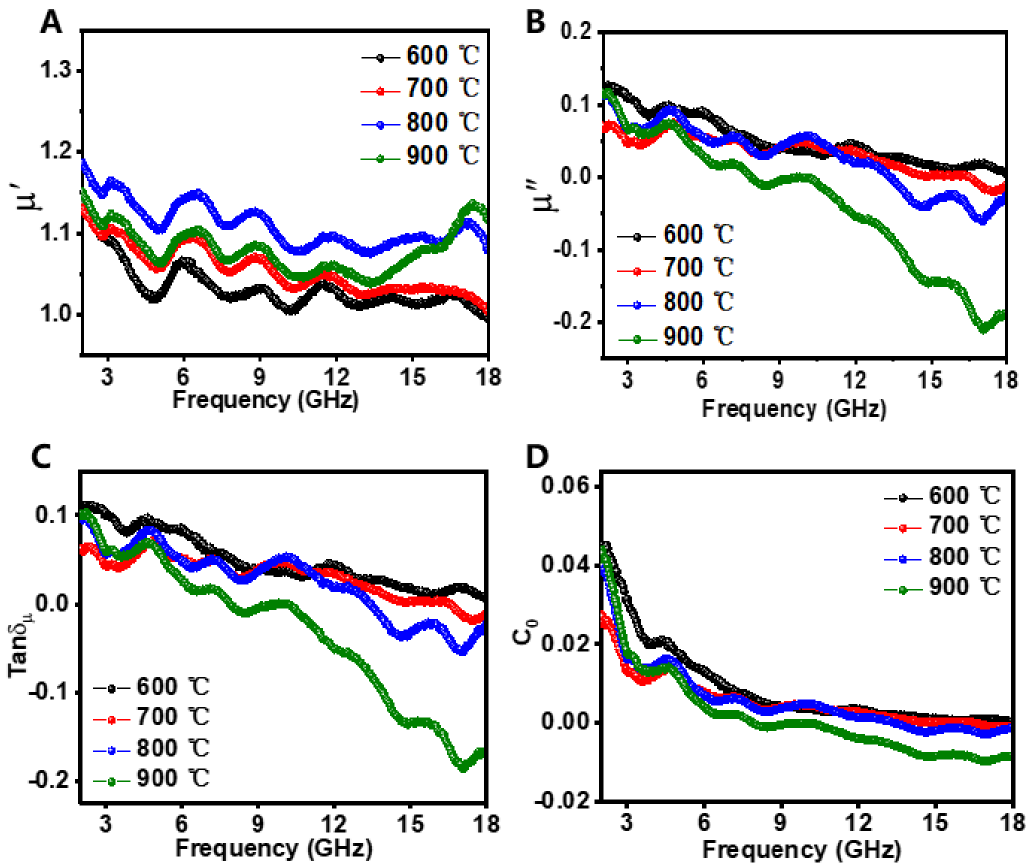

Fig. S22 (**A**) μ′, and (**B**) μ″parts of complex permeability, (**C**) Magnetic loss tangent $(\tan\delta_\mu = \frac{\mu''}{\mu'})$, (**D**) the eddy current values $(C_0 = \mu''(\mu')^{-2}f^{-1})$

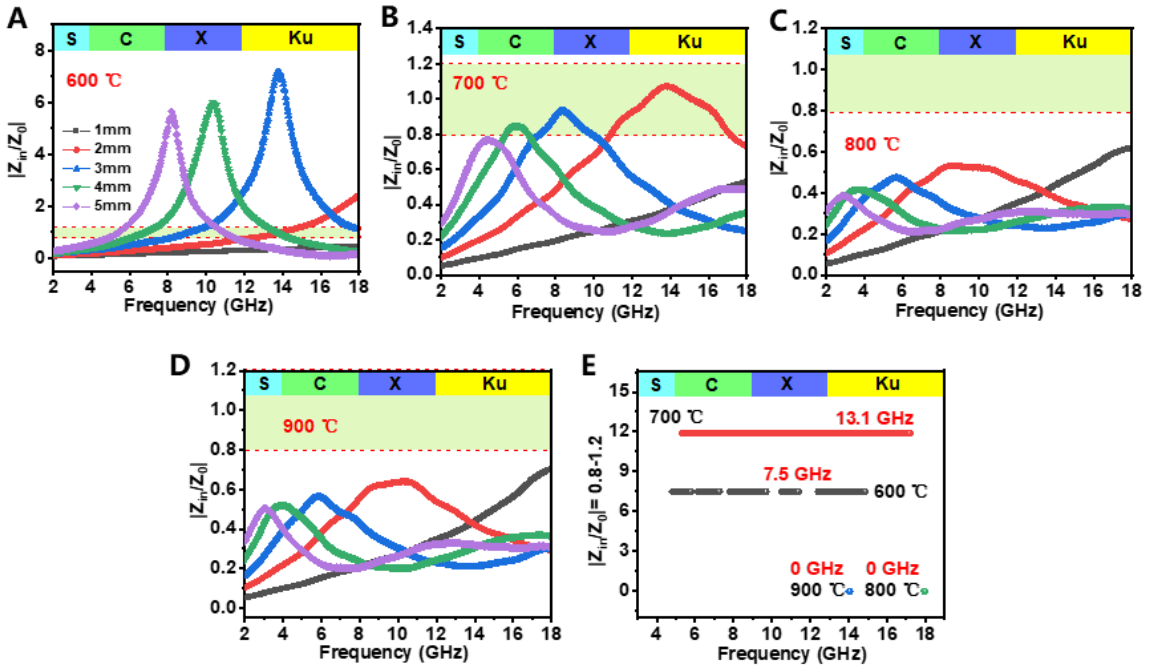

Fig. S23 The $|Z_{in}/Z_0|$ values with thicknesses in the range of 1–5 mm for (**A**) T600, (**B**) T700, (**C**) T800, and (**D**) T900, (**E**) The total frequency broad of $|Z_{in}/Z_0|$ values in the range of 0.8-1.2 with different thickness

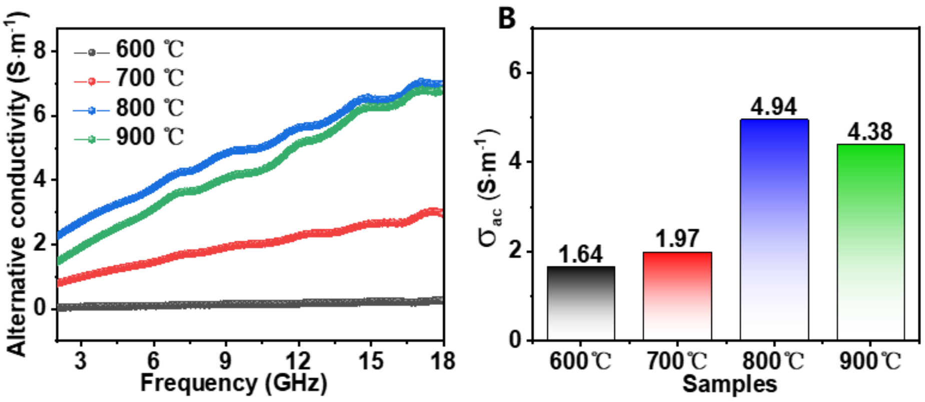

Fig. S24 (**A**) Alternative conductivity (σ_{ac}), and (**B**) the corresponding average conductivity

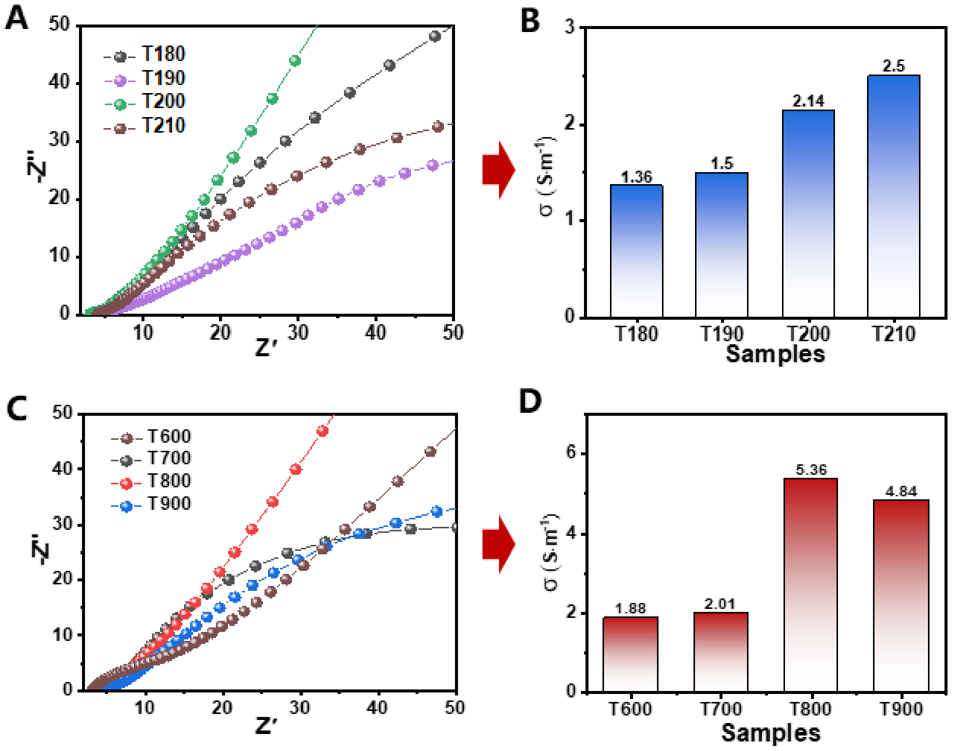

Fig. S25 (**A, B**) Nyquist plots, and (**B, D**) the corresponding conductivity calculated from Nyquist plots of different air@G-Fe/C samples

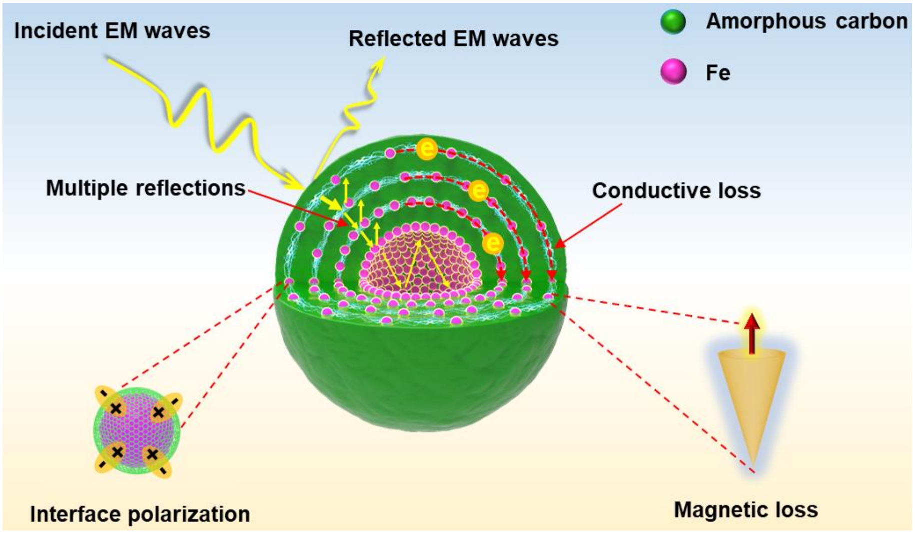

Fig. S26 Schematic of wave absorption mechanism of air@G-Fe/C nanospheres

Supplementary References

[S1] X. Zhou, Z. Jia, A. Feng, X. Wang, J. Liu et al., Synthesis of fish skin-derived 3D carbon foams with broadened bandwidth and excellent electromagnetic wave absorption performance. Carbon **152**, 827-836 (2019). https://doi.org/10.1016/j.carbon.2019.06.080

[S2] H. Lv, Z. Yang, H. Xu, L. Wang, R. Wu, An electrical switch-driven flexible electromagnetic absorber. Adv. Funct. Mater. **30**(4), 1907251 (2020). https://doi.org/10.1002/adfm.201907251

[S3] S. Dai, Y. Cheng, B. Quan, X. Liang, W. Liu et al., Porous-carbon-based Mo_2C nanocomposites as excellent microwave absorber: a new exploration. Nanoscale **10**(15), 6945-6953 (2018). https://doi.org/10.1039/C8NR01244J

[S4] Y. Wang, X. Han, P. Xu, D. Liu, L. Cui et al., Synthesis of pomegranate-like Mo_2C@C nanospheres for highly efficient microwave absorption. Chem. Eng. J. **372**, 312-320 (2019). https://doi.org/10.1016/j.cej.2019.04.153

[S5] Y. Guo, X. Jian, L. Zhang, C. Mu, L. Yin et al., Plasma-induced FeSiAl@Al_2O_3@SiO_2 core–shell structure for exceptional microwave absorption and anti-oxidation at high temperature. Chem. Eng. J. **384**, 123371 (2020). https://doi.org/10.1016/j.cej.2019.123371

[S6] S. Zhao, L. Yan, X. Tian, Y. Liu, C. Chen et al., Flexible design of gradient multilayer nanofilms coated on carbon nanofibers by atomic layer deposition for enhanced microwave absorption performance. Nano Res. **11**(1), 530-541 (2018). https://doi.org/10.1007/s12274-017-1664-6

[S7] M. Chen, Y. Zhu, Y. Pan, H. Kou, H. Xu et al., Gradient multilayer structural design of CNTs/SiO_2 composites for improving microwave absorbing properties. Mater. Des. **32**(5), 3013-3016 (2011). https://doi.org/10.1016/j.matdes.2010.12.043

[S8] B. Ji, S. Fan, S. Kou, X. Xia, J. Deng et al., Microwave absorption properties of multilayer impedance gradient absorber consisting of $Ti_3C_2T_x$ MXene/polymer films. Carbon **181**, 130-142 (2021). https://doi.org/10.1016/j.carbon.2021.05.018

[S9] Y. Zhao, X. Zuo, Y. Guo, H. Huang, H. Zhang et al., Structural engineering of hierarchical aerogels comprised of multi-dimensional gradient carbon nanoarchitectures for highly efficient microwave absorption. Nano-Micro Lett. **13**, 144 (2021). https://doi.org/10.1007/s40820-021-00667-7

[S10] C. Jin, Z. Wu, R. Zhang, X. Qian et al., 1D electromagnetic-gradient hierarchical carbon microtube via coaxial electrospinning design for enhanced microwave absorption. ACS Appl. Mater. Interfaces **13**(13), 15939-15949 (2021). https://doi.org/10.1021/acsami.1c03129

[S11] X.P. Li, Z. Deng, Y. Li, H.B. Zhang, S. Zhao et al., Controllable synthesis of hollow microspheres with Fe@ carbon dual-shells for broad bandwidth microwave absorption. Carbon **147**, 172-181 (2019). https://doi.org/10.1016/j.carbon.2019.02.073

[S12] F. Pan, Z. Liu, B. Deng, Y. Dong, X. Zhu et al., Lotus leaf-derived gradient hierarchical porous C/MoS_2 morphology genetic composites with wideband and tunable electromagnetic absorption performance. Nano-Micro Lett. **13**, 43 (2021). https://doi.org/10.1007/s40820-020-00568-1
